# Network meta-analysis of metformin, probiotics, krill oil, celecoxib, and glucosamine/chondroitin in the treatment of knee osteoarthritis

**DOI:** 10.3389/fmed.2026.1862817

**Published:** 2026-07-22

**Authors:** Keye Chen, Yan Sun, Longkang Cui, Yuchen Zhu, Bingbing Zhang, Haigang Wang

**Affiliations:** The Second Affiliated Hospital of Zhejiang Chinese Medical University, Hangzhou, China

**Keywords:** celecoxib, glucosamine combined with chondroitin, knee osteoarthritis, krill oil, metformin, network meta-analysis, probiotics

## Abstract

**Purpose:**

Knee osteoarthritis (KOA) creates a substantial global socioeconomic burden. Celecoxib remains a standard pharmacological treatment, but its long-term safety profile raises concerns. Several adjuvant therapies—probiotics, metformin, krill oil, and glucosamine/chondroitin (CS+GH)—have produced encouraging results in individual trials; however, direct head-to-head comparisons are absent. This network meta-analysis (NMA) compares the efficacy and safety of these five interventions to inform individualized treatment decisions in clinical practice.

**Methods:**

RCTs published from database inception through February 2026 were retrieved from PubMed, Web of Science, Embase, and the Cochrane Library. A random-effects NMA was conducted using Stata 18.0 and R (MetaInsight). Continuous outcomes were synthesized as mean differences (MD) and dichotomous safety endpoints as risk ratios (RR). Interventions were ranked by surface under the cumulative ranking curve (SUCRA), and evidence quality was assessed using the GRADE framework.

**Results:**

Twenty-three RCTs met the inclusion criteria. No significant network inconsistency (*P* > 0.05) or publication bias was detected. For analgesic efficacy, celecoxib ranked highest on VAS pain improvement (MD = −0.48, 95% CI −0.78 to −0.17, SUCRA = 0.93) and WOMAC total scores (SUCRA = 0.912), followed by CS+GH (0.756). Probiotics (0.66) and metformin (0.63) showed potential efficacy, but the final network certainty for most of these auxiliary comparisons was low or very low, mainly due to severe imprecision and the absence of direct head-to-head trials. These SUCRA-based rankings should therefore be interpreted cautiously in clinical settings. On WOMAC subscales, celecoxib was associated with the largest improvements in pain and physical function; krill oil ranked highest on the pain subscale (SUCRA = 0.99), while probiotics and metformin had the most favorable safety profiles (SUCRA = 0.885 and 0.724, respectively).

**Systematic Review Registration:**

https://www.crd.york.ac.uk/prospero/display_record.php?ID=CRD420261345598, identifier: CRD420261345598.

## 1 Introduction

### 1.1 Background and significance

Knee osteoarthritis (KOA) is characterized by the slow, progressive degradation of articular cartilage, subchondral bone sclerosis, and chronic synovitis. Since 1990, the global rise in population aging and obesity prevalence has caused steady increases in both the incidence and prevalence of KOA (1). KOA is now a leading cause of pain, functional limitation, and reduced health-related quality of life among middle-aged and older adults worldwide, imposing substantial economic costs on patients' families and healthcare systems alike.

Celecoxib, a highly selective cyclooxygenase-2 (COX-2) inhibitor, remains a first-line pharmacological treatment for KOA in both domestic and international clinical guidelines, owing to its anti-inflammatory and analgesic effects (1, 9, 17). However, long-term NSAID use—including celecoxib—carries well-documented risks of gastrointestinal mucosal injury and cardiovascular events, which sharply limits its applicability in high-risk populations such as the elderly and patients with pre-existing gastrointestinal or cardiovascular disease (29).

Recent advances in KOA pathogenesis research have progressively clarified mechanisms such as the “metabolic-inflammatory axis” and the “gut-joint axis.” These developments have drawn clinical attention to several emerging adjuvant therapies with distinct mechanisms of action. Metformin, a classic antidiabetic agent, can suppress the NF-κB-mediated inflammatory cascade via AMPK pathway activation (5, 6, 16) while also regulating chondrocyte autophagy to slow joint degeneration. Probiotics may reduce systemic low-grade inflammation by modulating the gut microbiota, indirectly alleviating joint inflammation and pain (3, 7, 11, 13, 15). Krill oil, rich in omega-3 polyunsaturated fatty acids and astaxanthin, offers combined anti-inflammatory and chondroprotective effects (8, 12, 21, 22). Glucosamine combined with chondroitin (CS+GH), a classic chondroprotective formulation, may improve joint lubrication and slow the breakdown of the cartilage extracellular matrix (2, 9, 14, 17, 18). Each of these therapies has been supported by multiple RCTs demonstrating symptomatic improvement in KOA patients.

The five intervention categories in this study span the four principal clinical routes for treating KOA: anti-inflammation and analgesia, chondroprotection, metabolic drug therapy, and gut microbiota modulation. These represent the most evidence-supported and commonly prescribed oral regimens for KOA, making it clinically meaningful to evaluate them within a single evidence network. Despite the growing diversity of treatment options, important gaps remain in the evidence base for clinical practice. Existing studies largely compare single interventions against placebo; long-term head-to-head trials across different mechanisms of action or drug classes are scarce. There is also no clear consensus on how different interventions perform relative to one another across distinct clinical endpoints such as pain relief and functional improvement. Prior NMAs on KOA have focused primarily on single drug classes—NSAIDs or chondroprotectants (27)—and no study has simultaneously compared the mid-term efficacy and safety of these five cross-pathway interventions. This absence of direct comparative evidence hinders the development of optimal, individualized long-term treatment strategies matched to a patient's specific symptom profile and baseline status.

We therefore conducted a systematic search for RCTs from database inception to February 2026 and performed a frequency-weighted NMA using Stata 18.0. We evaluated the efficacy and safety of five interventions—celecoxib, glucosamine plus chondroitin sulfate (CS+GH), krill oil, probiotics, and metformin—and ranked them by SUCRA scores. The 6-month follow-up was chosen as the primary endpoint because it captures the stable mid-term efficacy of oral agents while minimizing dropout and intervention-switching bias that often affect ultra-long-term follow-up data. This time point represents a standard benchmark in contemporary KOA pharmacotherapy trials. The study aims to address gaps in the current evidence and supply updated data to support multimodal, individualized, and longer-term management of KOA.

## 2 Materials and methods

This network meta-analysis was prospectively registered in the International Prospective Register of Systematic Reviews (PROSPERO) with registration number: **CRD420261345598** [PROSPERO: CRD420261345598].

This study was conducted and reported in full compliance with the PRISMA-NMA 2020 (Priority Reporting Items for Systematic Reviews and Meta-Analyses of Network Meta-Analyses) guidelines. All information was obtained exclusively from published sources; the study involved no participant privacy concerns or ethical issues, and therefore neither ethics committee approval nor participant informed consent was required.

### 2.1 Data collection

#### 2.1.1 Inclusion criteria

1. Study Design: Published RCTs regardless of blinding.
2. Study Population: Adult patients who met international clinical diagnostic criteria for KOA [e.g., as determined by the American College of Rheumatology (ACR) or Osteoarthritis Research Society International (OARSI)], without restrictions based upon gender, stage of illness, or degree of severity of the illness.
3. Interventions: The intervention in the experimental group was any one of the following five categories: celecoxib, glucosamine combined with chondroitin (CS+GH), krill oil, probiotics, or metformin. The control group received a placebo or any one of the five aforementioned interventions as a positive control, with no restrictions on dosage, frequency, or duration of administration.
4. Outcome Measures: the total score for the Western Ontario and McMaster Universities Osteoarthritis Index (WOMAC), the WOMAC pain subscale score, or the WOMAC function subscale score; the incidence of adverse events reported by the authors in their publication were also analyzed as a safety end point.

#### 2.1.2 Exclusion criteria

1. Non-randomized controlled studies, including reviews, systematic reviews, conference abstracts, case reports, animal studies, and cell studies;
2. Studies on secondary KOA (e.g., post-traumatic arthritis, osteoarthritis secondary to rheumatoid arthritis, etc.);
3. Studies where the intervention entailed the concomitant use of two or more study medications and the effects of individual agents could not be isolated;
4. Studies with absent outcome data such that full effect estimates and 95% confidence intervals cannot be derived and for which the necessary information remains unobtainable despite contacting the original investigators;
5. Works released in any language except Chinese and English.

### 2.2 Literature search strategy

We electronically searched PubMed, Web of Science, Embase, and The Cochrane Library from database inception to February 2026. A Boolean retrieval approach was employed using both controlled vocabulary (MeSH/Emtree) and free-text keywords. The search terms included “Knee Osteoarthritis,” “Knee OA,” “Osteoarthritis of the Knee,” “Probiotics,” “Metformin,” “Krill oil,” “Celecoxib,” “Glucosamine,” “Chondroitin,” “Randomized Controlled Trial,” and “RCT.”

### 2.3 Literature screening and data extraction

Two reviewers with formal standardized training independently performed study screening and data extraction according to prespecified inclusion and exclusion criteria. The screening process consisted of three sequential stages: (1) deduplication of records, (2) title and abstract screening to exclude clearly irrelevant studies, and (3) full-text assessment of the remaining publications to finalize the included set.

The following information was extracted: (1) basic study characteristics—lead author, publication year, country, study design, and follow-up duration; (2) participants' baseline characteristics—sample size per arm, age, sex, and disease severity; (3) intervention and control protocols—drug type, dose, frequency, treatment duration, and control group details; and (4) outcome data—mean ± SD of VAS pain scores, WOMAC total scores, and subscale scores at 6-month follow-up, along with adverse event counts.

Disagreements during screening and data extraction were first resolved through joint re-examination of the original reports and group discussion; if consensus could not be reached, an independent expert in evidence-based medicine was consulted for a final decision. When published data were incomplete, the corresponding author was contacted by email to request the missing information; studies for which data remained unavailable were excluded.

For multi-arm trials with three or more intervention groups, data extraction followed prespecified rules: when multiple arms belonged to the same intervention category, sample sizes, means, and SDs were pooled using sample-size-weighted formulas; when arms represented distinct node interventions, data from all relevant arms were extracted. For studies with multiple arms sharing a common comparator, we used Stata's multivariate network meta-analysis framework, which appropriately models correlated effect sizes and prevents double-counting of the shared comparator arm.

### 2.4 Assessment of risk of bias in included studies

Two independent reviewers assessed the risk of bias in each included study using the Cochrane Collaboration's Risk of Bias 2.0 (RoB 2.0) tool for randomized controlled trials, covering the five standard domains: (1) risk of bias arising from the randomization process, (2) risk of bias due to deviations from intended interventions, (3) risk of bias due to missing outcome data, (4) risk of bias in measurement of the outcome, and (5) risk of bias in selection of the reported result. Each domain was rated as low risk of bias, some concerns, or high risk of bias. Disagreements were resolved through team discussion or consultation with external experts.

### 2.5 Statistical analysis methods

Software of statistical calculation for network meta-analysis was Stata 18.0:

1. Effect size estimation: Continuous endpoints (e.g., VAS and WOMAC scale scores) were synthesized using mean difference (MD) with 95% CI, while binary safety endpoints (e.g., incidence of adverse events) were summarized as risk ratio (RR) with 95% CI.
2. Analytical approach: Given expected differences across studies in baseline participant profiles, intervention dose, and treatment duration, a random-effects framework was applied to all analyses. Between-study heterogeneity was evaluated using the I^2^ statistic with its 95% confidence interval (CI). Heterogeneity was classified as follows: I^2^ ≤ 25%, low; 25% < I^2^ < 50%, moderate; I^2^ ≥ 50%, substantial. I^2^ values are reported alongside their 95% CIs in the results.
3. Efficacy and safety ranking: Interventions were quantitatively ranked by surface under the cumulative ranking curve (SUCRA) scores. A SUCRA score closer to 1 (100%) indicates a higher probability that the intervention is among the most effective (or safest) for the given outcome.
4. Consistency assessment: A node-splitting procedure was used to evaluate concordance between direct and indirect evidence, with a significance threshold of α = 0.05. *P* > 0.05 indicates no statistically significant inconsistency, in which case the consistency model was retained; otherwise, the inconsistency model was applied and the source of discrepancy was examined. Loop inconsistency testing provided supplementary verification for closed loops within the evidence network.
  Sensitivity analysis: Leave-one-out sensitivity analysis was performed for each outcome by iteratively removing one study at a time and re-estimating the NMA model under the random-effects framework. The stability of pooled effect estimates was assessed by comparing leave-one-out results with full-network estimates. Additionally, given variability in methodological quality across studies, network estimates were recalculated after excluding studies rated as “some concerns” or “high risk” under RoB 2.0 to evaluate the robustness of the primary findings.
5. Assessment for publication bias: WOMAC function subscale scores—selected because this domain contained the largest number of included studies and the best statistical power—were used to construct funnel plots for visual inspection of potential publication bias.
6. Grading of evidence quality: We applied the Grading of Recommendations Assessment, Development, and Evaluation (GRADE) framework to assess the certainty of evidence for each key comparison in the NMA. Evidence certainty was classified as high, moderate, low, or very low. Downgrading criteria included study limitations, indirectness, inconsistency, imprecision, and publication bias.
7. To properly account for multi-arm trials, a multivariate random-effects NMA framework was employed. The network package in Stata 18.0 and R (MetaInsight) automatically calculated and adjusted for the correlation and covariance across contrasts derived from the same multi-arm study. This approach ensures that each multi-arm trial contributes appropriate weight to the evidence network exactly once, preventing inflation of sample sizes and unit-of-analysis errors.

### 2.6 Reporting standards

This study adhered strictly to the PRISMA-NMA 2020 statement. Key outcomes—including study flow, evidence network diagrams, pooled effect sizes, and risk-of-bias judgments—were presented using standardized tables and figures with full labels, titles, and annotations. Supplementary files provide all original data and analysis procedures to enable replication and verification of the results.

## 3 Results

### 3.1 Literature search outcomes and baseline features of the studies included

The initial database search identified 890 records. After removing 221 duplicates, 669 citations remained for screening. Title and abstract screening excluded 600 clearly irrelevant records, leaving 69 for full-text retrieval and eligibility assessment. Following full-text review, 46 studies were excluded for not meeting the inclusion criteria: 18 due to study design; 16 because of issues with the study population, intervention, or comparator; and 12 owing to insufficient or non-extractable outcome data.

**Figure 1:**
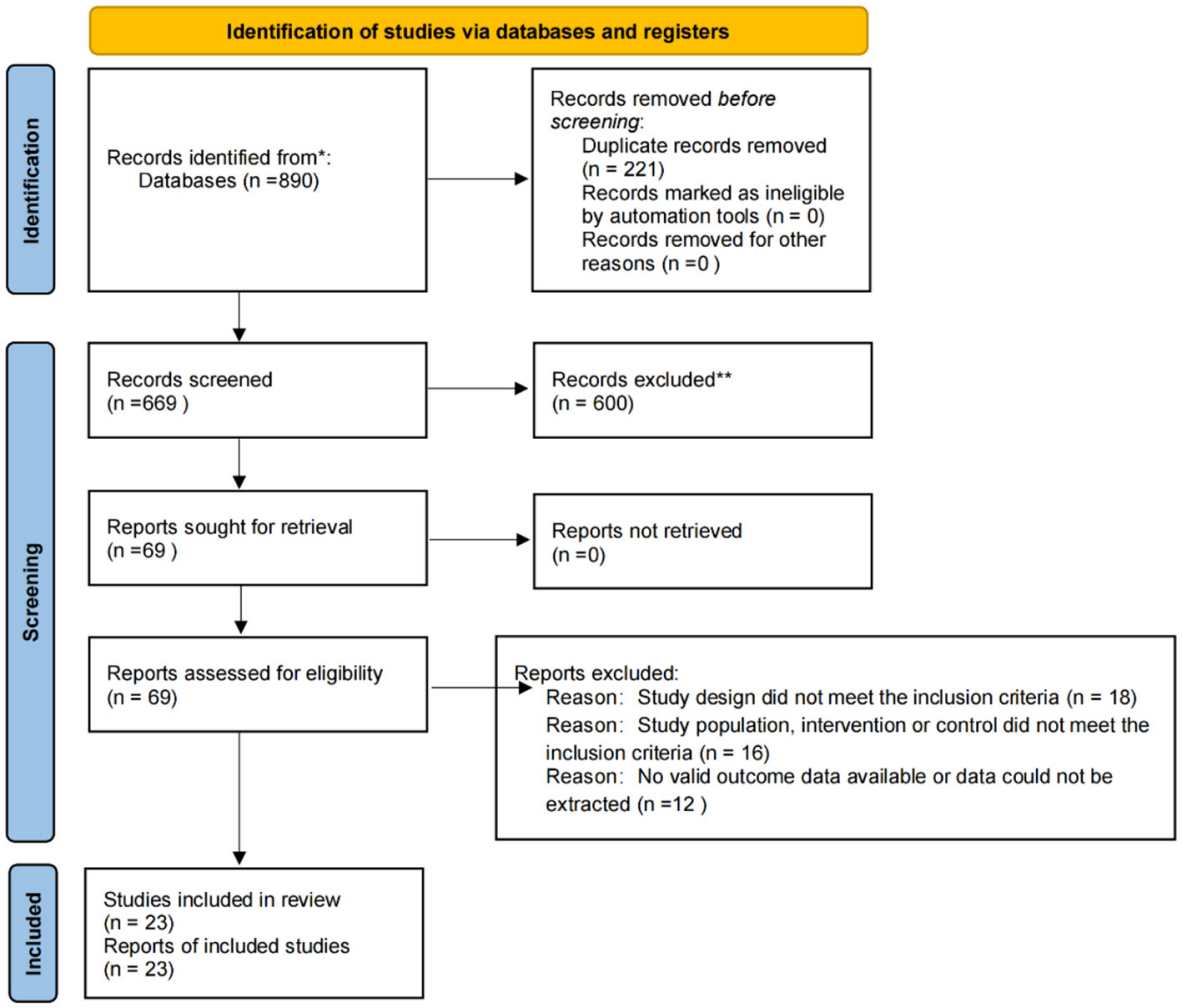
Literature search outcomes.

Ultimately, 23 RCTs that met all criteria were included. Figure 1 (PRISMA 2020 screening flowchart) illustrates the literature screening process.

Across the 23 included RCTs, patients in the intervention and control arms had comparable baseline characteristics. All trials reported age, sex, and disease duration at baseline; detailed baseline data are summarized in Table 1.

**Table 1:** Baseline features of the studies included.

References	Country	Intervention	Comparator	Sample size (I/C)	Age (Mean±SD)
Lubis et al. (2017)	Indonesia	GlcN 1,500 mg + CS 1,200 mg + MSM 500 mg, qd	GlcN 1,500 mg + CS 1,200 mg; Placebo	50/39	58.3 ± 10.4
Hochberg et al. (9)	France, Germany, Poland, Spain^*^	CS 400 mg + GlcN 500 mg, tid	Celecoxib 200 mg, qd	264/229	62.2 ± 8.8
Gibofsky et al. (1)	United States, Canada^*^	Celecoxib 200 mg, qd	Rofecoxib 25 mg, qd; Placebo	189/130	62.2 ± 10.5
Shine et al. (20)	Republic of Korea	Latilactobacillus sakei LB-P12 350 mg/day (10^10^ CFU), qd	Placebo	50/45	61.34 ± 8.31
Clegg et al. (2)	United States^*^	GlcN 500 mg, tid	Placebo; CS 400 mg tid; Celecoxib 200 mg qd	317/199	58.6 ± 10.2
Laslett et al. (12)	Australia^*^	Krill Oil 2 g/day (EPA 380 mg + DHA 200 mg)	Placebo	130/66	61.7 ± 9.3
Stonehouse et al. (21)	Australia^*^	Krill Oil 4 g/day (EPA 600 mg + DHA 280 mg + Astaxanthin 450 μg)	Placebo	117/65	55.8 ± 6.8
Suzuki et al. (22)	Japan	Krill Oil 2 g/day (EPA 240 mg + DHA 110 mg)	Placebo	25/21	65.8 ± 10.1
Alimoradi et al. (6)	Iran	Metformin Hydrochloride 0.5 g−1.5 g/day, titrated	Placebo	44/34	49.3 ± 9.3
Pan et al. (16)	Australia	Metformin Sustained-Release 2,000 mg/day, titrated	Placebo	54/38	57.8 ± 9.5
Sawitzke et al. (19)	United States^*^	GlcN 500 mg, tid	Placebo; CS 400 mg tid; Celecoxib 200 mg qd	134/92	56.7 ± 10.7
Lyu et al. (15)	Taiwan, China	Streptococcus thermophilus TC1633 2 × 10^9^ CFU/day, qd	Placebo	37/31	65.1 ± 9.3
Karim et al. (10)	Pakistan	Multistrain Probiotic Vivomix 112 billion CFU/day	Placebo	46/49	69.3 ± 5.5
Karim et al. (11)	United Arab Emirates, Pakistan^*^	Multistrain Probiotic Vivomix 112 billion CFU/day	Placebo	65/45	69.3 ± 5.5
Dolatkhah et al. (7)	Iran	Saccharomyces boulardii 5 × 10^9^ CFU/day, qd	Placebo	32/20	56.70 ± 9.05
Lei et al. (13)	China	Lactobacillus casei Shirota 2 × 10^9^ CFU/day	Placebo	215/120	66.5 ± 5.2
Bensen et al. (1999)	United States, Canada^*^	Celecoxib 50 mg/100 mg/200 mg, bid	Placebo; Naproxen 500 mg bid	203/349	62 ± 12.3
Nannoni et al. (2020)	Italy	Verbascox^®^ Herbal Extract 800 mg/day	No treatment; Celecoxib 200 mg/day	50/38	57.1 ± 5.3
Roman-Blas et al. (18)	Spain^*^	CS 1,200 mg + GlcN Sulfate 1,500 mg, qd	Placebo	80/67	65 ± 8
Reginster et al. (17)	Belgium, Czech Republic, Italy, Poland, Switzerland^*^	Pharmaceutical-grade CS 800 mg/day	Placebo; Celecoxib 200 mg/day	199/156	65.5 ± 8.0
Karim et al. (11)	Pakistan	Probiotics (Vivomix 112 billion CFU/day), 12 weeks	Placebo	65/45	69.3 ± 5.5
Laslett et al. (12)	Australia^*^	Krill Oil 2 g/day (containing EPA 380 mg + DHA 200 mg)	Placebo (mixed vegetable oil, no EPA and DHA)	130/130	≥40 years (no mean ± SD reported)
Ruan et al. (2022)	China^*^	Metformin Hydrochloride Sustained-Release Tablets (0.5 g/day in weeks 1–2, 1 g/day in weeks 3–4, 2 g/day maintenance, 24 months)	Placebo (identical inert tablets containing microcrystalline cellulose, HPMC, magnesium stearate, lactose)	131/131	50–75 years (no mean ± SD reported)
Lubis et al. (2017)	Indonesia	GlcN 1,500 mg + CS 1,200 mg + MSM 500 mg, qd	GlcN 1,500 mg + CS 1,200 mg; Placebo	50/39	58.3 ± 10.4

### 3.2 Results of the risk of bias assessment

**Figure 2:**
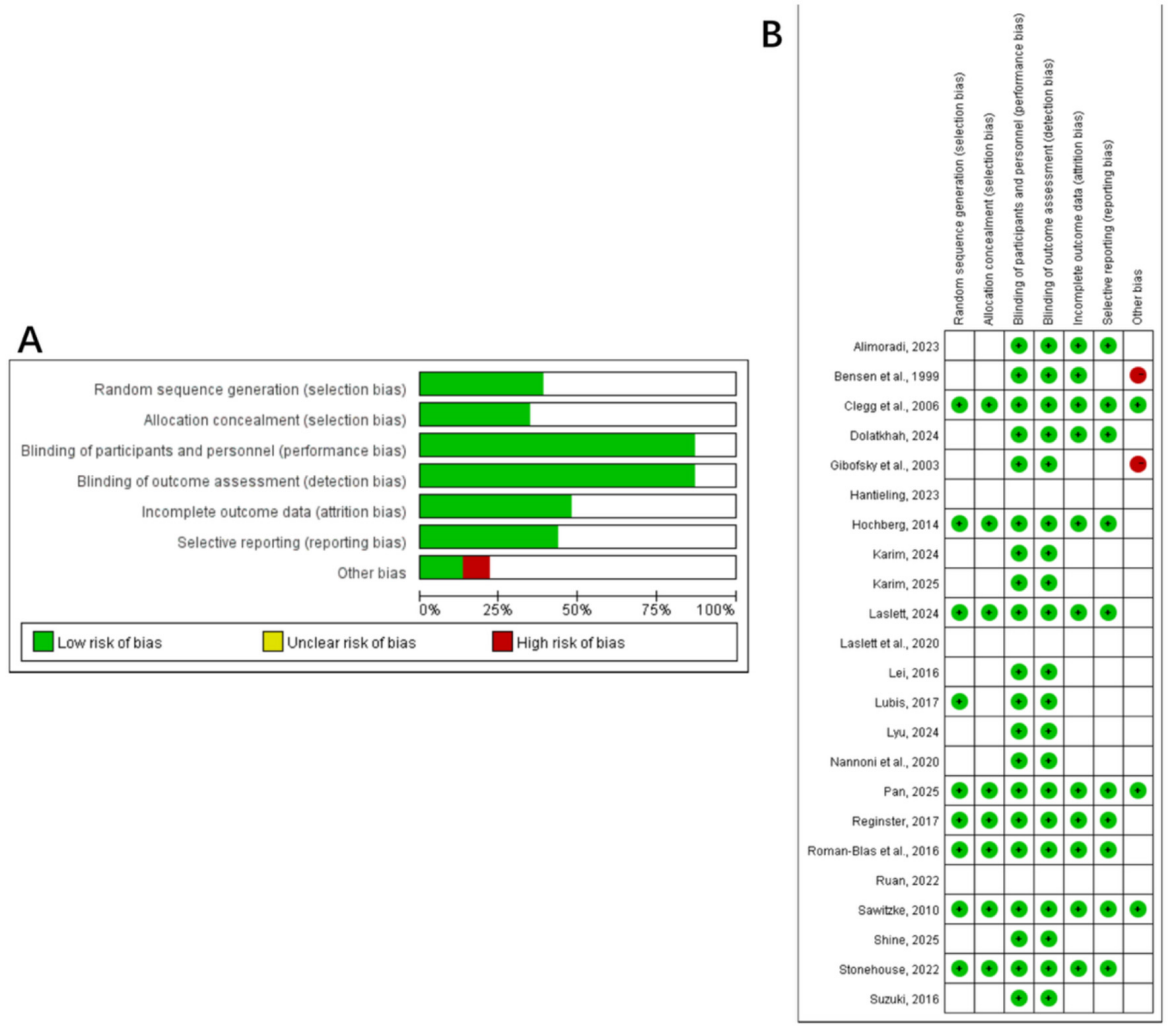
Summary of bias risk.

Most included studies were rated as low risk of bias in the random sequence generation and selective reporting domains, reflecting clear methodological descriptions. The allocation concealment and blinding domains showed a higher proportion of unclear or high-risk ratings, primarily due to insufficient reporting of concealment procedures and blinding protocols in several trials. Attrition bias was also a notable concern, with several studies rated as unclear or high risk because of incomplete outcome data or inadequate handling of missing values. The “other bias” domain had the highest proportion of high-risk judgments, driven by limited reporting of confounding variables and other potential sources of bias. A small number of studies were rated at high risk across two or more domains. Overall, the risk of bias was judged to be moderate, with key limitations concentrated in allocation concealment, blinding, and other bias (Figure 2).

### 3.3 Results of the network meta-analysis

#### 3.3.1 Consistency test and evidence network diagram

**Figure 3:**
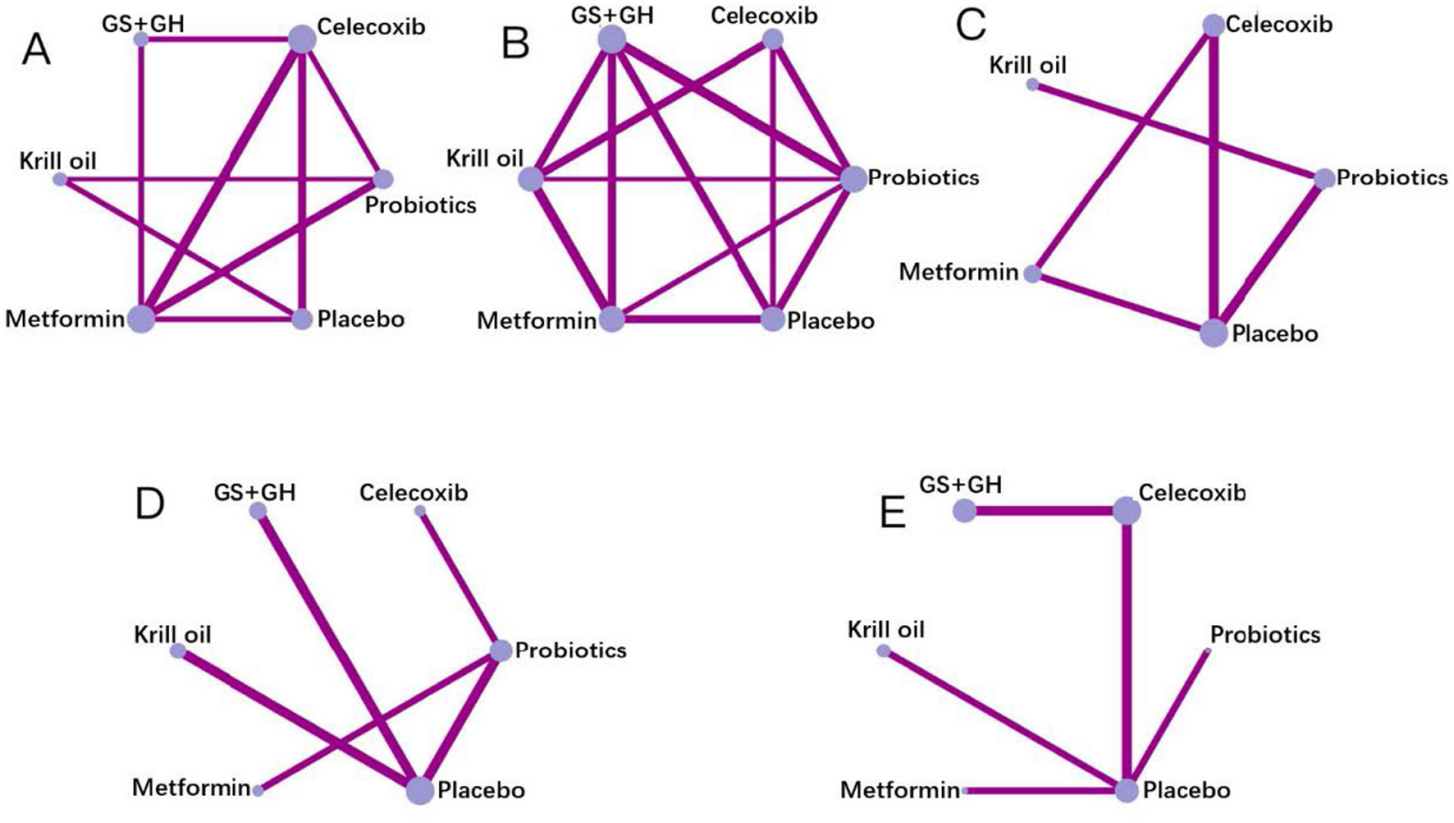
Figures network evidence: **(A)** WOMAC functional scores. **(B)** VAS pain scores. **(C)** WOMAC pain scores. **(D)** WOMAC total scores. **(E)** Safety outcome.

We constructed evidence networks and generated network plots for five outcomes: VAS pain scores, total WOMAC scores, WOMAC pain subscale scores, WOMAC function subscale scores, and adverse event incidence. All networks were loop-closed. Consistency analysis using the node-splitting method yielded *P* > 0.05 for all outcomes, indicating no statistically significant inconsistency between direct and indirect evidence in any network; a consistency model was therefore applied for all subsequent analyses. Figures 3A–E present the evidence network for each outcome. In those figures, the size of each node reflects the total number of patients randomized to each intervention, and the edge width between two intervention nodes denotes how many head-to-head RCTs are compared to these interventions.

#### 3.3.2 Analysis of efficacy outcomes

##### 3.3.2.1 VAS pain scores

Twelve RCTs reported VAS pain scores at 6-month follow-up in KOA patients, spanning 6 interventions (celecoxib, CS+GH, krill oil, probiotics, metformin, and placebo). NMA results showed that, compared with placebo, all active interventions demonstrated statistically significant differences in analgesic efficacy (*P* < 0.05). Among them, celecoxib provided the strongest analgesic effect, outperforming most other active interventions on both resting and post-activity pain. Detailed pooled effect estimates for VAS pain scores are presented in Supplementary File S1. Heterogeneity in direct comparisons varied: glucosamine vs. placebo (I^2^ = 98.1%, substantial; *p* < 0.001), krill oil vs. placebo (I^2^ = 41.3%, moderate; *p* = 0.192), and probiotics vs. placebo (I^2^ = 96.3%, substantial; *p* < 0.001).

**Figure 4:**
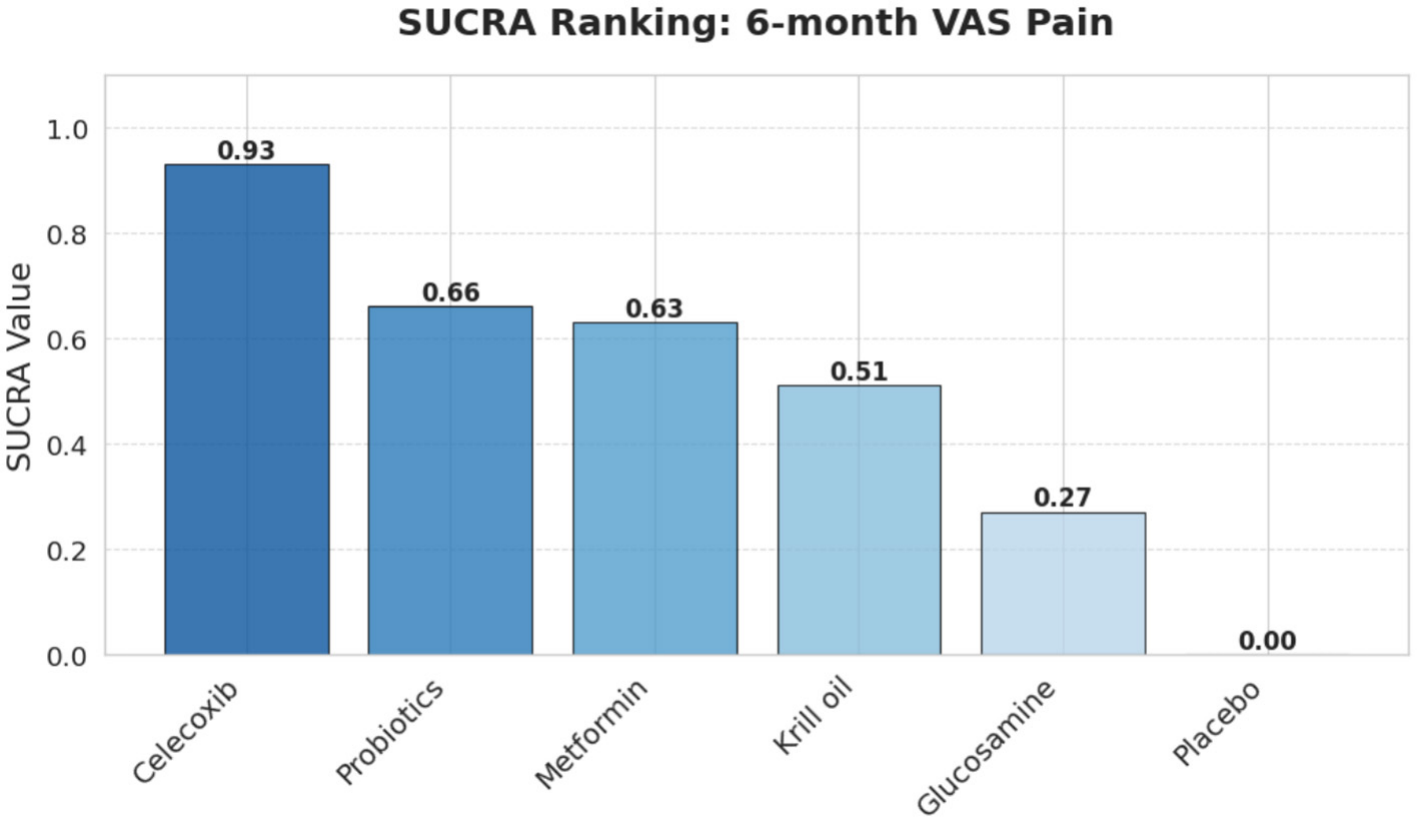
SUCRA ranking: 6-month VAS pain.

SUCRA rankings for VAS pain reduction were celecoxib (0.93) > probiotics (0.66) > metformin (0.63) > krill oil (0.51) > glucosamine (0.27) > placebo (0.00); the ranking results are shown in Figure 4.

##### 3.3.2.2 WOMAC total scores

Four RCTs reported WOMAC total scores at 6-month follow-up in KOA patients, involving six interventions. NMA results showed that, compared with placebo, all active interventions demonstrated statistically significant improvements in overall joint symptoms (*P* < 0.05); celecoxib produced the greatest overall improvement, followed by CS+GH. Detailed pooled effect estimates for the WOMAC total score are presented in Supplementary File S2. NMA-estimated effect sizes for key comparisons vs. placebo were celecoxib (MD = −0.97, 95% CI: −5.41 to 3.46), glucosamine (MD = −3.14, 95% CI: −5.75 to −0.53), krill oil (MD = −1.05, 95% CI: −3.62 to 1.52), and probiotics (MD = −0.67, 95% CI: −3.24 to 1.89). Heterogeneity in direct comparisons varied considerably: glucosamine vs. placebo (I^2^ = 98.1%, substantial; *p* < 0.001), krill oil vs. placebo (I^2^ = 41.5%, moderate; *p* = 0.192), and probiotics vs. placebo (I^2^ = 96.3%, substantial; *p* < 0.001).

**Figure 5:**
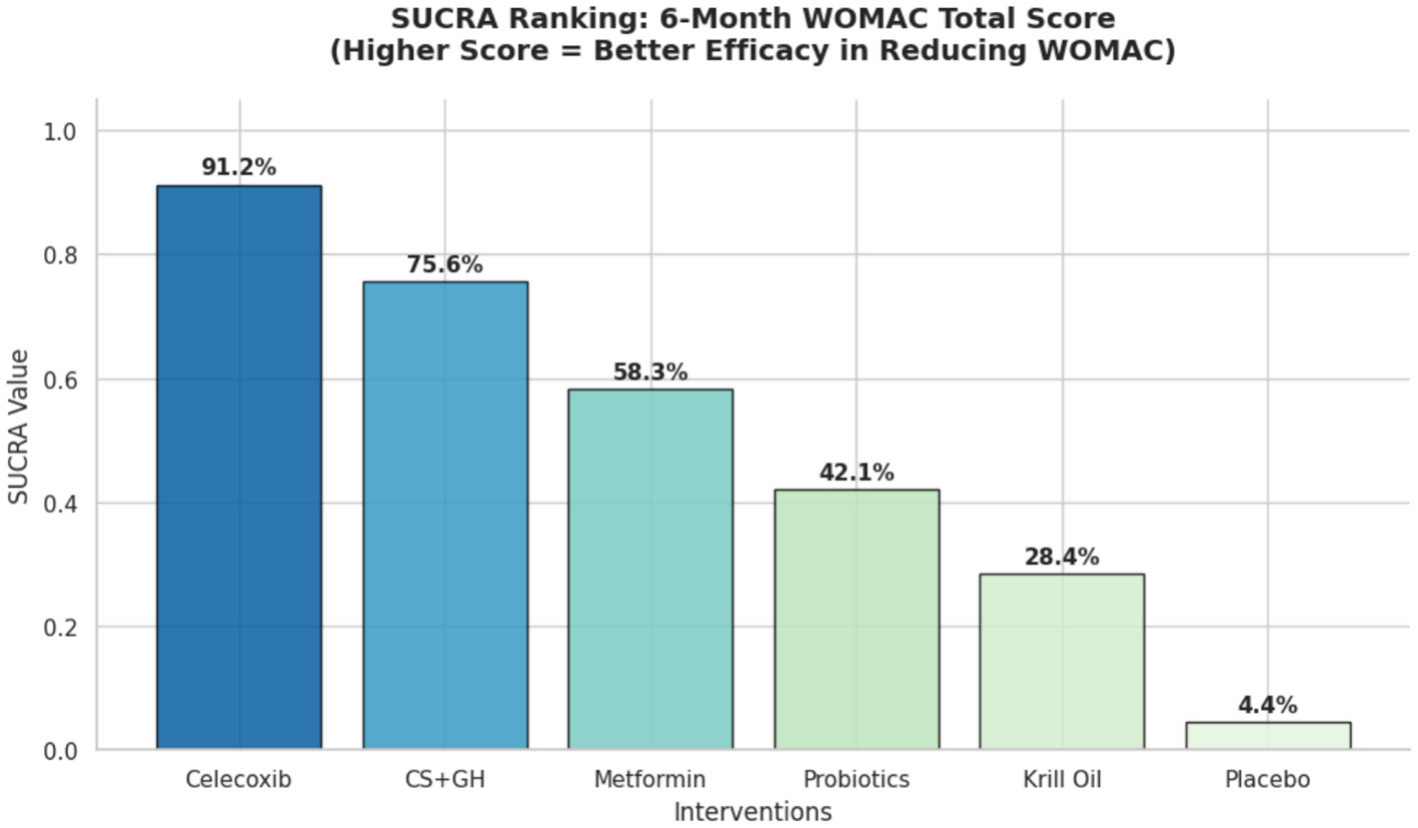
SUCRA ranking: 6-month WOMAC total scores.

SUCRA rankings for WOMAC total score improvement were celecoxib (91.2%) > CS+GH (75.6%) > metformin (58.3%) > probiotics (42.1%) > krill oil (28.4%) > placebo (4.4%). The ranking results are shown in Figure 5.

##### 3.3.2.3 WOMAC pain subscale scores

Five RCTs reported WOMAC pain subscale scores at 6-month follow-up in KOA patients, covering five interventions (krill oil, probiotics, metformin, celecoxib, and placebo). NMA results indicated that all active treatments differed significantly from placebo in joint pain relief (*P* < 0.05). Krill oil exhibited the strongest analgesic effect and was significantly superior to the other active treatments. Detailed pooled effect estimates for the WOMAC pain subscale are provided in Supplementary File S3. NMA-estimated effect sizes vs. placebo were krill oil (MD = −4.12, 95% CI: −5.61 to −2.63), metformin (MD = −1.36, 95% CI: −2.28 to −0.44), celecoxib (MD = −0.63, 95% CI: −1.38 to 0.13), and probiotics (MD = −2.62, 95% CI: −3.59 to −1.65). Heterogeneity in direct comparisons was observed: celecoxib vs. placebo (I^2^ = 88.8%, substantial; *p* = 0.003) and probiotics vs. placebo (I^2^ = 69.2%, moderate; *p* = 0.071).

**Figure 6:**
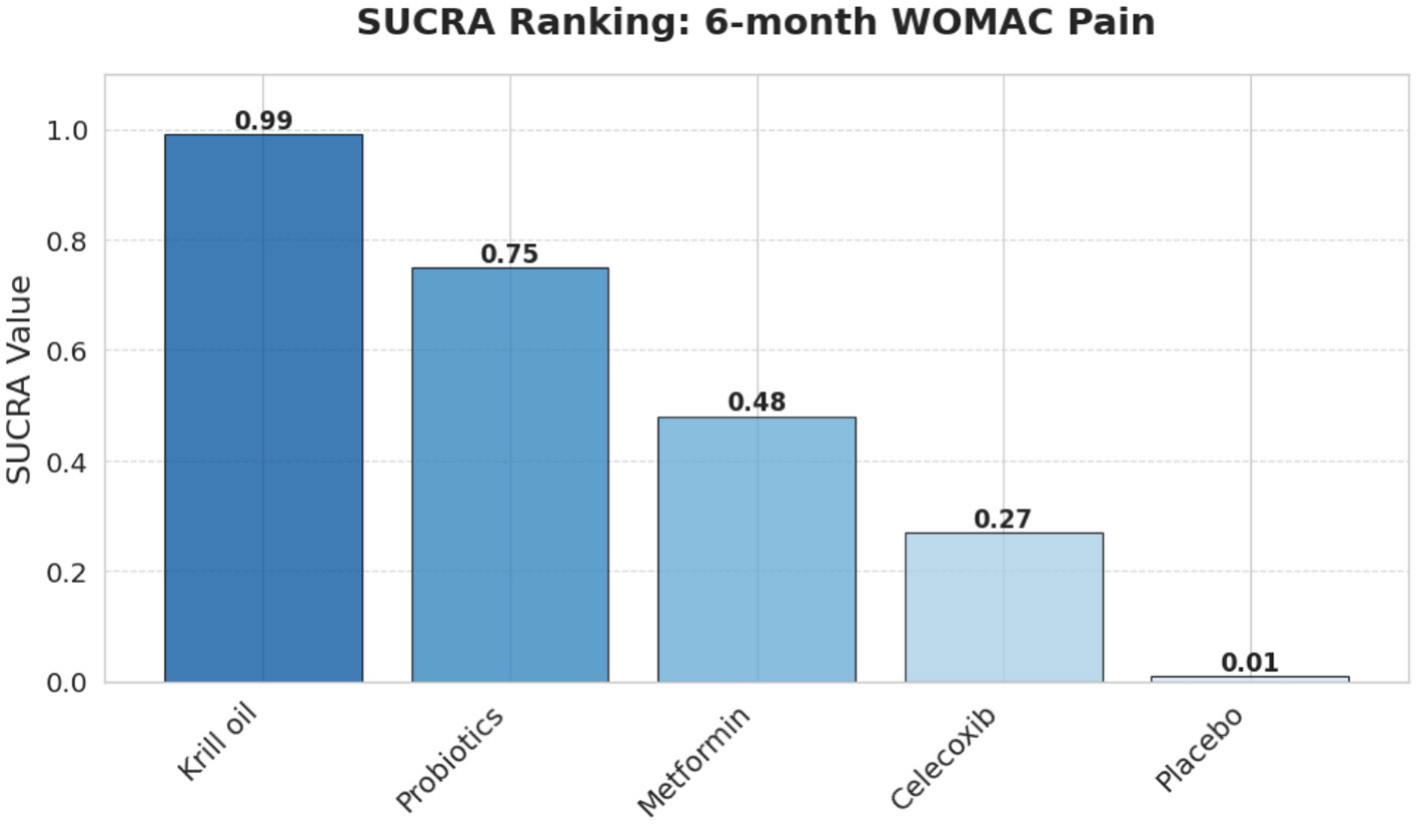
SUCRA ranking: 6-month WOMAC pain scores.

SUCRA rankings for WOMAC pain subscale improvement were krill oil (0.99) > probiotics (0.75) > metformin (0.48) > celecoxib (0.27) > placebo (0.01); the ranking results are shown in Figure 6.

##### 3.3.2.4 WOMAC functional subscale scores

Five RCTs reported WOMAC functional subscale scores at 6-month follow-up in KOA patients, involving six interventions. NMA results showed that, compared with placebo, krill oil, celecoxib, metformin, and probiotics all demonstrated statistically significant improvements in joint function (*P* < 0.05); celecoxib produced the greatest functional improvement. Detailed NMA results for the WOMAC functional subscale are provided in Supplementary File S4. NMA-estimated effect sizes vs. placebo were krill oil (MD = −1.67, 95% CI: −3.05 to −0.29), metformin (MD = −0.72, 95% CI: −1.66 to 0.22), celecoxib (MD = −0.90, 95% CI: −1.76 to −0.04), and glucosamine (MD = −0.86, 95% CI: −2.19 to 0.47). Heterogeneity in direct comparisons: metformin vs. celecoxib (I^2^ = 94.7%, substantial; *p* < 0.001), placebo vs. celecoxib (I^2^ = 0.0%, low; *p* = 0.787), and metformin vs. probiotics (I^2^ = 0.0%, low; *p* = 0.586).

**Figure 7:**
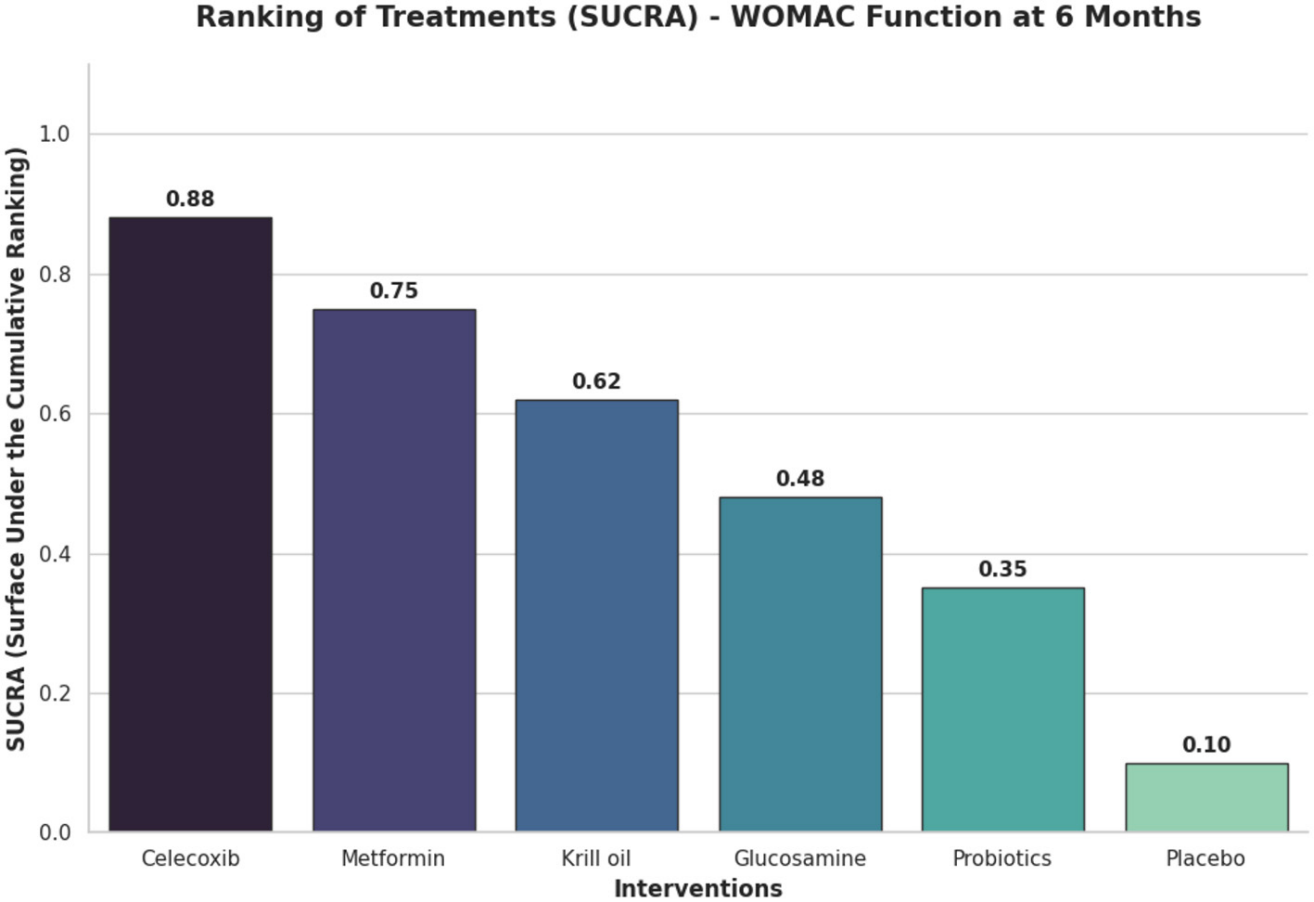
SUCRA ranking: 6-month WOMAC function.

SUCRA rankings for WOMAC functional subscale improvement were celecoxib (0.88) > metformin (0.75) > krill oil (0.62) > glucosamine (0.48) > probiotics (0.35) > placebo (0.10); see Figure 7 for the ranking results.

#### 3.3.3 Safety outcome analysis

**Figure 8:**
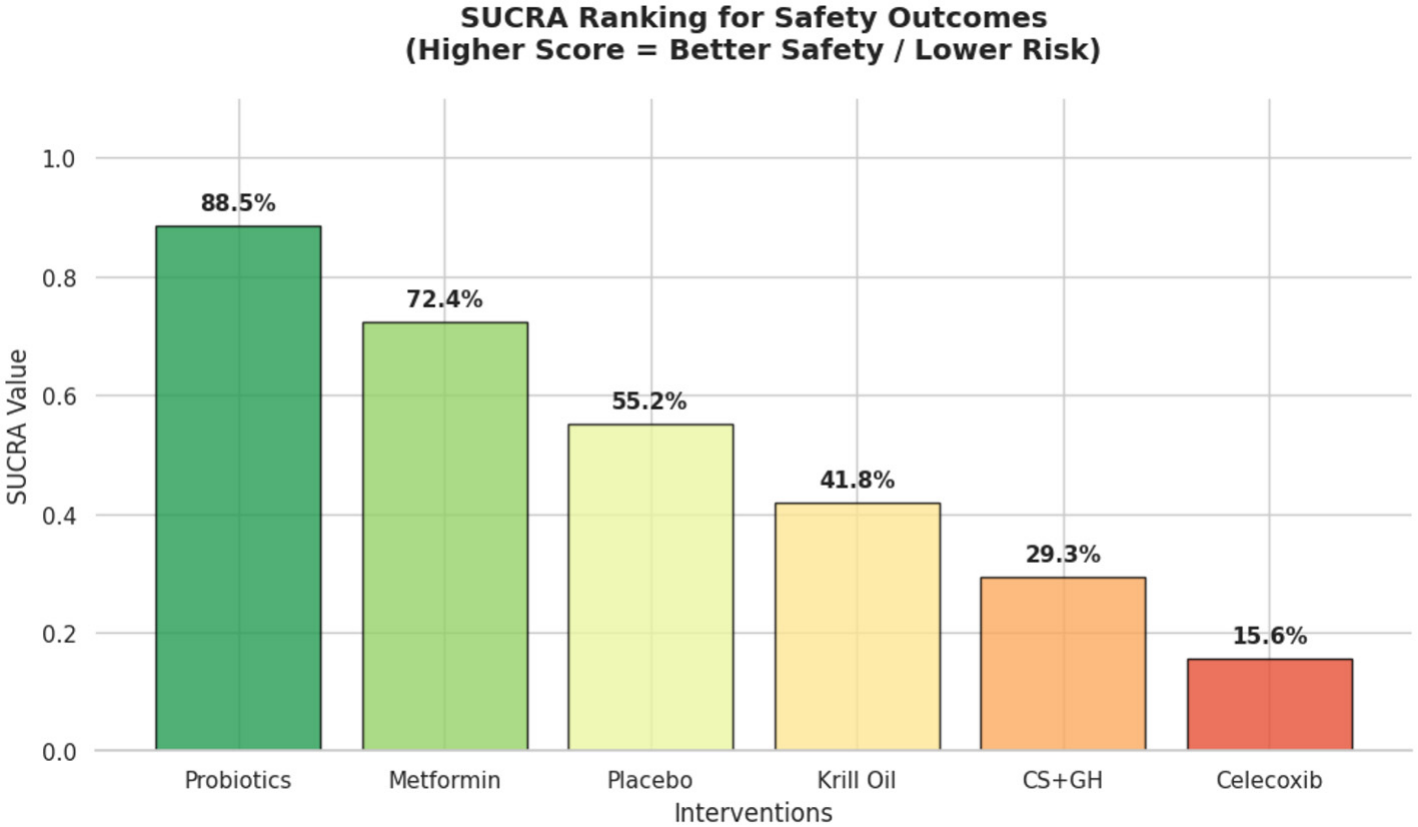
SUCRA ranking for safety outcomes.

Nine RCTs reported treatment-related adverse event (AE) incidence at 6 months in KOA patients across six intervention types. NMA did not reveal any statistically significant differences between interventions for the risk of adverse events (all *P* > 0.05). All interventions were well tolerated, with no serious treatment-related adverse events recorded. Detailed pooled effect estimates for safety outcomes are provided in Supplementary File S5. NMA-estimated risk ratios for key comparisons were celecoxib vs. placebo (RR = 1.38, 95% CI: 0.82 to 2.34), CS+GH vs. celecoxib (RR = 0.78, 95% CI: 0.45 to 1.36), metformin vs. placebo (RR = 1.53, 95% CI: 0.67 to 3.49), and probiotics vs. placebo (RR = 0.65, 95% CI: 0.10 to 4.06). Heterogeneity in direct safety comparisons were celecoxib vs. placebo (I^2^ = 53.9%, moderate; *p* = 0.141) and CS+GH vs. celecoxib (I^2^ = 71.9%, substantial; *p* = 0.059). SUCRA safety rankings were probiotics (88.5%) > metformin (72.4%) > placebo (55.2%) > krill oil (41.8%) > CS+GH (29.3%) > celecoxib (15.6%), indicating that probiotics and metformin have the most favorable overall safety profiles. The ranking results are shown in Figure 8.

#### 3.3.4 Sensitivity analysis

**Figure 9:**
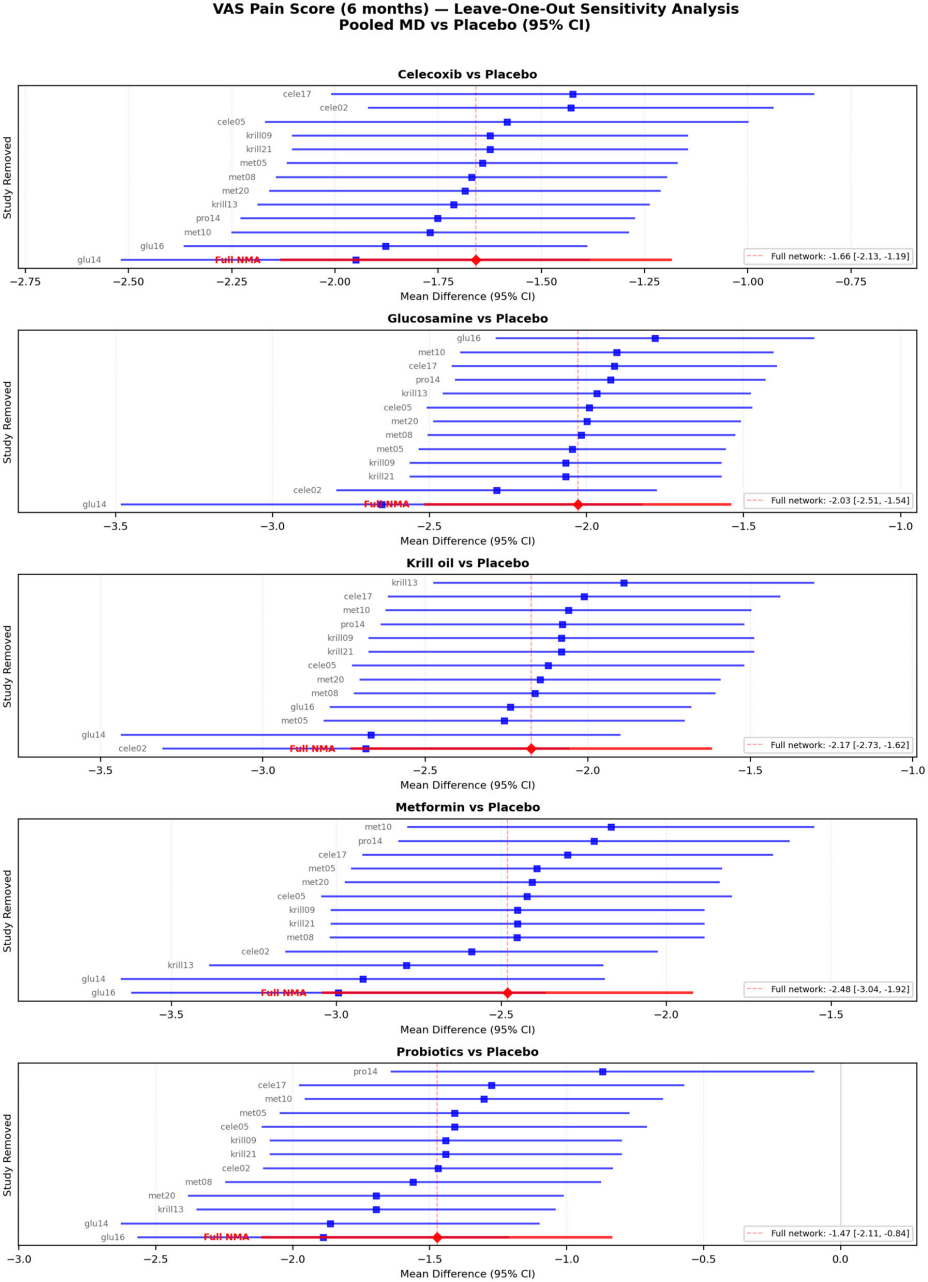
Leave-one-out sensitivity analysis for VAS pain scores at 6 months.

**Figure 10:**
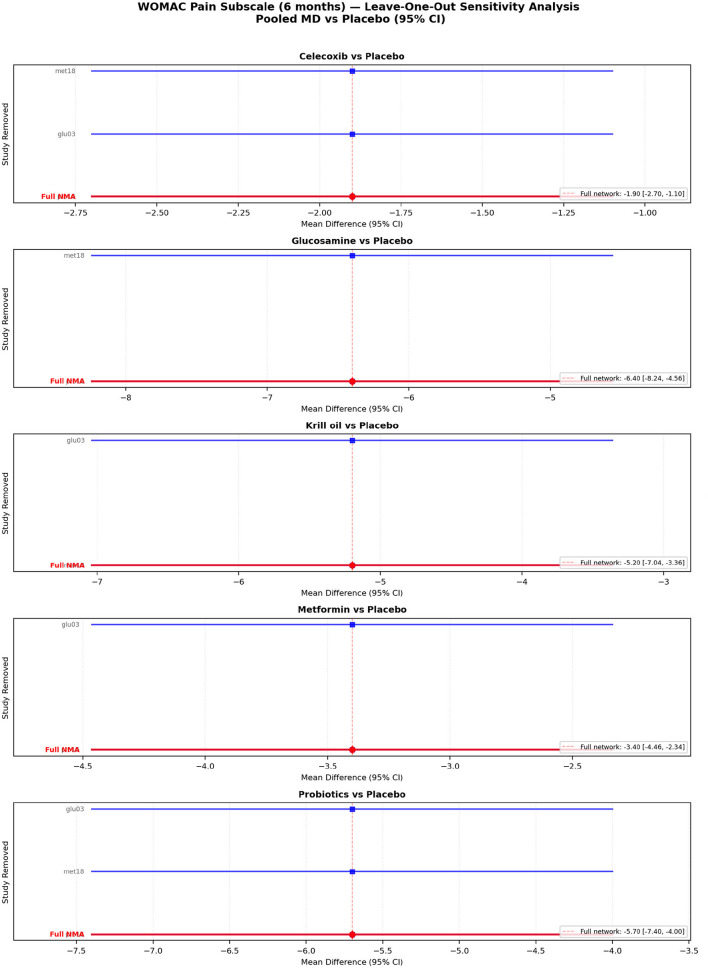
Leave-one-out sensitivity analysis for WOMAC pain subscale scores at 6 months.

**Figure 11:**
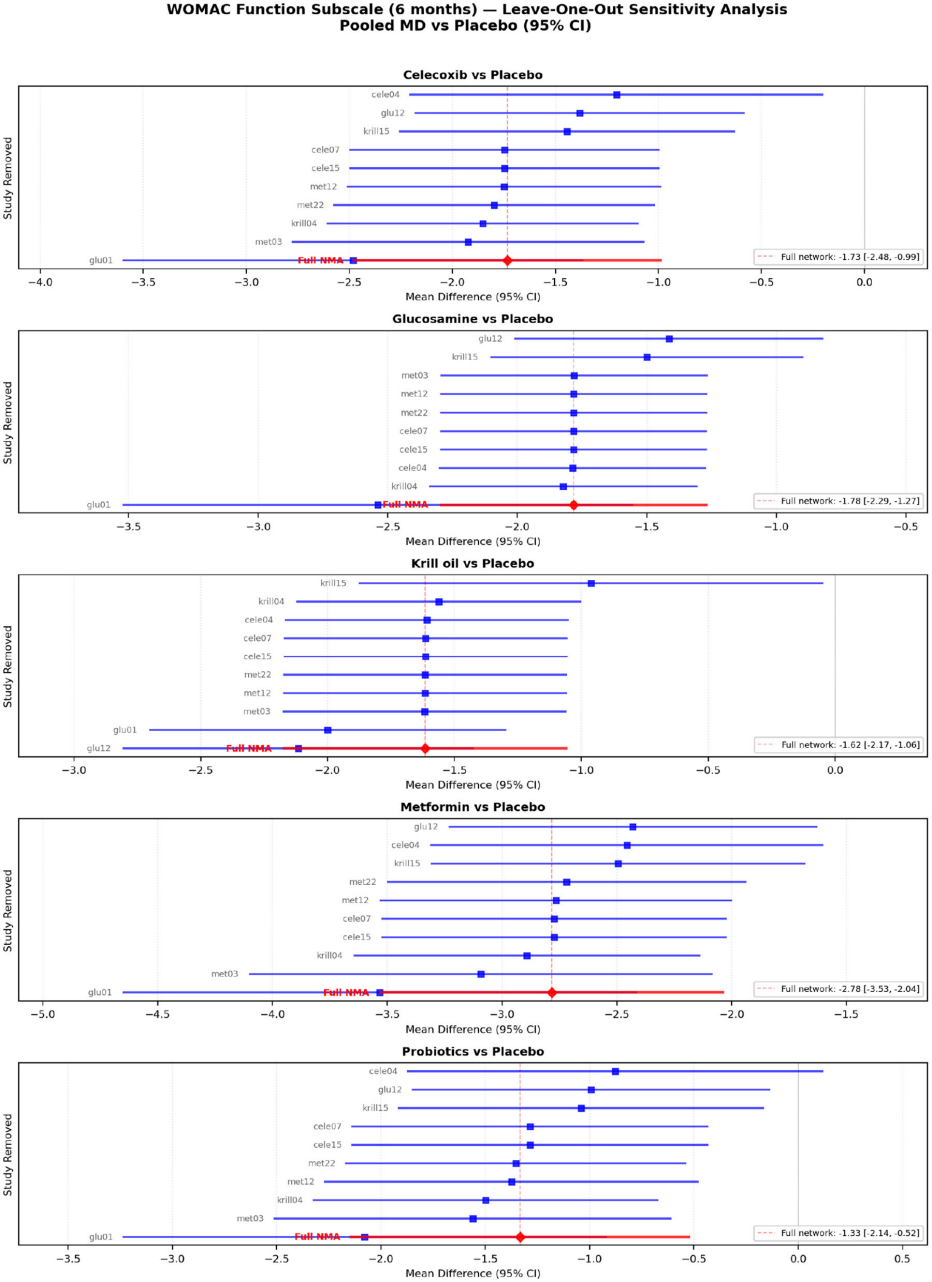
Leave-one-out sensitivity analysis for WOMAC functional subscale scores at 6 months.

Leave-one-out sensitivity analyses showed that network effect estimates were generally robust across all five outcomes (Figures 9–11). For VAS pain scores (12 RCTs), the direction and statistical significance of all treatment effects relative to placebo remained unchanged after sequential exclusion of each study, with a maximum absolute deviation of |Δ| = 0.11 in the pooled effect estimate Figure 9. For WOMAC total scores, the glucosamine vs. placebo comparison changed from statistically significant to non-significant upon removal of one study (18), indicating that this comparison was driven by limited data; all other comparisons remained stable. For WOMAC pain scores, the metformin vs. placebo comparison lost statistical significance when one study was removed, and the celecoxib vs. placebo comparison gained significance after removal of another, reflecting the sparse evidence base (five RCTs, with only two direct comparisons containing multiple studies). For WOMAC functional scores, significance changes were observed in several comparisons when individual studies were removed, consistent with the limited number of studies per direct comparison. Safety outcome results were stable across all iterations. Overall, the network estimates for the largest outcome network (VAS pain) were highly robust, while the instability observed in WOMAC subscale analyses underscores the need for additional direct evidence Figures 10, 11 to confirm these findings.

### 3.4 Results of publication bias analysis

**Figure 12:**
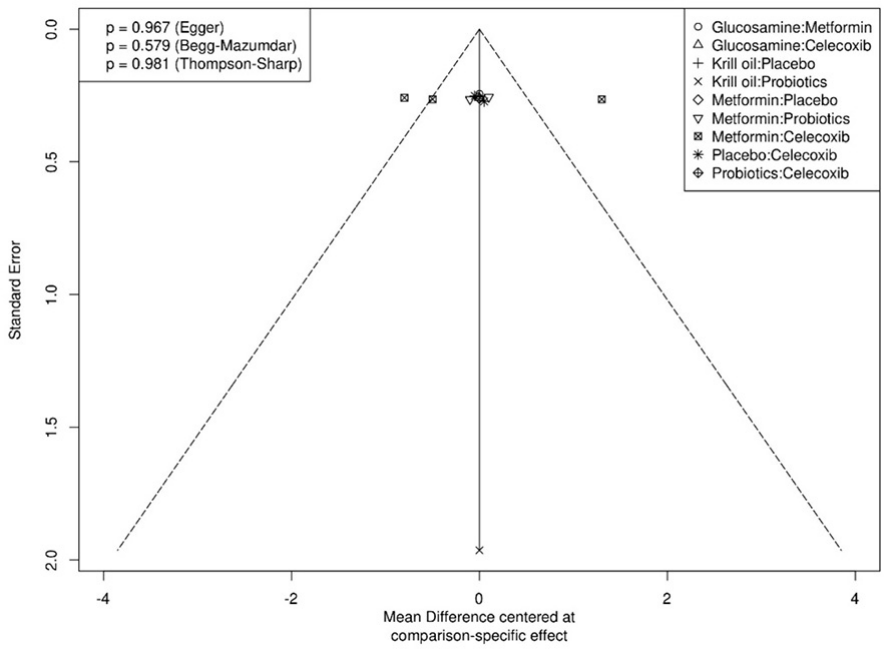
Results of publication bias analysis.

We used the WOMAC functional subscale score—the outcome with the largest number of included studies and the best statistical power—to construct a funnel plot for assessing publication bias (Figure 12). The scatter points for each study were symmetrically distributed around the pooled effect size, with no outliers significantly exceeding the 95% CI. Quantitative analysis confirmed an extremely low likelihood of publication bias: Egger's test (*P* = 0.967), Begg–Mazumdar test (*P* = 0.579), and Thompson–Sharp test (*P* = 0.981) all yielded *P* > 0.05, indicating that the results are well-supported and not driven by selective publication.

### 3.5 Grading of evidence quality

We applied the NMA-specific GRADE framework to evaluate the certainty of evidence separately for direct, indirect, and final network estimates for the primary comparisons; the findings are presented in Table 2. Only three comparisons achieved moderate-quality evidence (celecoxib vs. metformin, celecoxib vs. probiotics, and krill oil vs. placebo), each downgraded one level for serious imprecision. The remaining 12 comparisons were rated as very-low-quality evidence—driven by serious imprecision, very serious inconsistency (I^2^ > 90% for glucosamine vs. placebo and probiotics vs. placebo), and reliance on indirect evidence where direct RCTs were unavailable. The higher of the direct or indirect evidence ratings served as the starting point for each network estimate and could be further downgraded. Methodological appraisal via Cochrane RoB 2.0 indicated a generally acceptable bias profile: most included studies had low risk in random sequence generation and selective reporting, while moderate concerns were identified in allocation concealment, blinding, attrition, and other bias domains, with a minority of studies judged at high risk in at least one domain. Publication bias was not suggested across any comparison. Consequently, “Risk of Bias” and “Publication Bias” were rated as “not serious” for all comparisons (Figure 2).

**Table 2:** GRADE evidence profile for network meta-analysis of interventions.

Comparison (Arm 1 vs. Arm 2)	Evidence type	No. of studies	Sample size	Effect size [95% CI]	Risk of bias	Inconsistency	Indirectness	Publication bias	Imprecision	Certainty/ GRADE	Summary of reasons
**Celecoxib vs. Glucosamine**	Indirect	0	150	2.50 [1.75; 3.25]	Not serious	Not suggested	Not serious	Not serious	Serious	Very low	Only indirect evidence; indirect effective sample size (0) did not meet OIS (800); downgraded due to imprecision.
**Celecoxib vs. Krill oil**	Indirect	0	139	0.09 [−0.52; 0.69]	Not serious	Not suggested	Not serious	Not serious	Serious	Very low	Only indirect evidence; 95% CI crosses the null line (0), high imprecision. (Note: This result indicates no statistically significant difference between krill oil and celecoxib.)
**Celecoxib vs. Metformin**	Indirect	0	88	0.70 [0.17; 1.23]	Not serious	Not suggested	Not serious	Not serious	Serious	Moderate	Only indirect evidence; sample size below OIS (800), downgraded one level for imprecision. However, the initial indirect rating was very high, thus maintaining moderate certainty.
**Celecoxib vs. Placebo**	Indirect	0	326	−0.97 [−1.44; −0.49]	Not serious	Not suggested	Not serious	Not serious	Serious	Very low	Only indirect evidence; indirect effective sample size (154) below OIS (800); downgraded due to imprecision.
**Celecoxib vs. Probiotics**	Direct	1	229	−0.30 [−0.68; 0.08]	Not serious	Not serious	Not serious	Not serious	Serious	Moderate	Direct study had no significant bias or heterogeneity, but 95% CI crosses the null line (0); downgraded one level for imprecision.
**Glucosamine vs. Krill oil**	Indirect	0	201	−2.41 [−3.10; −1.72]	Not serious	Not suggested	Not serious	Not serious	Serious	Very low	Only indirect evidence; sample size (191) did not meet OIS (800); downgraded due to imprecision.
**Glucosamine vs. Metformin**	Indirect	0	150	−1.80 [−2.55; −1.05]	Not serious	Not suggested	Not serious	Not serious	Serious	Very low	Only indirect evidence; sample size too small and did not meet OIS (800); downgraded due to imprecision.
**Glucosamine vs. Placebo**	Direct	2	388	−3.47 [−4.04; −2.89]	Not serious	Very serious	Not serious	Not serious	Serious	Very low	The two included direct studies had extremely high heterogeneity (I^2^ = 98.1%); downgraded two levels for inconsistency; sample size below 800; downgraded one level for imprecision.
**Glucosamine vs. Probiotics**	Indirect	0	291	−2.80 [−3.45; −2.15]	Not serious	Not suggested	Not serious	Not serious	Serious	Very low	Only indirect evidence; indirect effective sample size (212) did not meet OIS (800); downgraded due to imprecision.
**Krill oil vs. Metformin**	Indirect	0	139	0.61 [0.01; 1.22]	Not serious	Not suggested	Not serious	Not serious	Serious	Very low	Only indirect evidence; sample size severely insufficient and below 800; downgraded due to imprecision.
**Krill oil vs. Placebo**	Direct	2	377	−1.05 [−1.43; −0.68]	Not serious	Not serious	Not serious	Not serious	Serious	Moderate	Direct evidence quality acceptable; heterogeneity acceptable (I^2^ = 41.3%). Total sample size (377) below default OIS (800); downgraded one level for imprecision.
**Krill oil vs. Probiotics**	Indirect	0	280	−0.39 [−0.86; 0.09]	Not serious	Not suggested	Not serious	Not serious	Serious	Very low	Only indirect evidence; 95% CI crosses the null line (0), high imprecision; downgraded to very low.
**Metformin vs. Placebo**	Indirect	0	326	−1.67 [−2.14; −1.19]	Not serious	Not suggested	Not serious	Not serious	Serious	Very low	Only indirect evidence; indirect effective sample size (154) below OIS (800); downgraded due to imprecision.
**Metformin vs. Probiotics**	Direct	1	229	−1.00 [−1.38; −0.62]	Not serious	Not serious	Not serious	Not serious	Serious	Moderate	Includes direct RCT evidence with no significant bias. Sample size (229) below OIS; downgraded one level for imprecision.
**Probiotics vs. Placebo**	Direct	2	467	−0.67 [−0.96; −0.37]	Not serious	Very serious	Not serious	Not serious	Serious	Very low	Includes 2 direct RCT studies, but heterogeneity was extremely severe (I^2^ = 96.3%); downgraded two levels for inconsistency; sample size below 800; downgraded one level for imprecision.

Risk of bias for all comparisons was rated as “not serious” because quality assessment via the Cochrane RoB 2.0 tool indicated that the majority of the included randomized controlled trials (RCTs) presented low risk of bias or some concerns, with no single study heavily weighted as high risk.

The final network evidence certainty ranged from moderate to very low, mainly limited by statistical incoherence, inconsistency, and severe imprecision.

Moderate-certainty network evidence: Only three final network estimates (celecoxib vs. metformin, celecoxib vs. probiotics, and krill oil vs. placebo) achieved moderate certainty. These were downgraded by one level primarily due to imprecision, as their cumulative optimal information size (OIS) was suboptimal or their 95% CIs approached the clinical null line. Notably, while the direct estimate for krill oil vs. placebo was relatively stable (I^2^ = 41.3%), its network estimate was slightly penalized by indirect evidence propagation.

Low-to-very-low-certainty network evidence: The remaining 12 final network estimates fell into lower certainty tiers; their clinical rankings should therefore be interpreted with extreme caution. The network estimates for glucosamine vs. placebo and probiotics vs. placebo were graded as very low certainty, driven by extreme statistical heterogeneity in their direct evidence (I^2^ = 98.1% and 96.3%, respectively). For other cross-intervention comparisons lacking direct head-to-head evidence (e.g., metformin vs. krill oil and probiotics vs. glucosamine), the final network estimates relied entirely on indirect loops and were uniformly rated as very low certainty due to the compound penalties of indirectness and marked imprecision (wide 95% CIs).

## 4 Discussion

### 4.1 Key findings

This network meta-analysis compared the efficacy and safety of five KOA treatments with distinct mechanisms of action at 6-month follow-up. It should be noted that 12 of the 15 final network estimates were graded as low or very low certainty (GRADE; Table 2), meaning that the SUCRA-based rankings discussed below should be interpreted as provisional, not definitive. With this caveat, the analysis suggested differentiated clinical strengths across interventions: celecoxib ranked highest on the SUCRA for general pain control, total symptom improvement, and functional recovery (1, 9, 17, 23); krill oil ranked highest on the WOMAC pain subscale (8, 12, 21, 22); and probiotics and metformin received the most favorable safety rankings and AE rates comparable to placebo (5, 7, 10, 11, 16, 28). Consistency testing and publication bias analyses showed acceptable network behavior, but the GRADE assessment—dominated by serious imprecision and inconsistency—indicates that most comparative rankings rest on limited evidence.

### 4.2 Interpretation of study results and discussion of mechanisms

SUCRA rankings placed celecoxib as the intervention most likely to rank first for VAS pain, WOMAC total scores, and WOMAC functional subscale improvement, although the evidence supporting its superiority over other active interventions was limited by sparse direct head-to-head data. As a highly selective COX-2 inhibitor, celecoxib blocks the arachidonic acid metabolic pathway, reducing prostaglandin synthesis and rapidly suppressing synovial inflammation (1, 29). This analgesic action enables patients to engage more fully in daily activities and rehabilitation, which in turn drives improvements in physical function scores. The finding is consistent with KOA clinical guidelines—both domestic and international—that position NSAIDs as first-line agents for symptom management. Although NSAIDs are not thought to have direct chondroprotective effects, their capacity to interrupt the “pain-inactivity” cycle makes them valuable for short-to-medium-term functional recovery in KOA.

Krill oil ranked first on the WOMAC pain subscale (SUCRA = 0.99) and showed competitive results on the functional subscale (SUCRA = 0.62). Given that direct head-to-head evidence involving krill oil was limited, these rankings should be weighed accordingly. Two mechanisms may explain this pattern. First, its abundant omega-3 polyunsaturated fatty acids competitively inhibit cyclooxygenase and lipoxygenase (LOX) pathways, producing a mild but sustained anti-inflammatory effect (8, 21, 27). Second, its natural astaxanthin content provides potent antioxidant activity that can inhibit matrix metalloproteinases (MMPs) and reduce chondrocyte apoptosis (8, 12, 21). Although the immediate impact on physical function may be less pronounced than that of potent NSAIDs such as celecoxib, these structural properties suggest that krill oil may offer advantages in long-term disease modification and joint preservation, making it a reasonable alternative for patients seeking non-pharmacological or adjunctive treatment.

On the safety side, probiotics and metformin received the highest SUCRA safety rankings (rank 1 and 2 respectively), both with significantly fewer AEs than celecoxib and AE rates comparable to placebo (5, 7, 10, 11, 16, 26, 28); however, most safety comparisons were based on indirect evidence and wide confidence intervals, limiting the certainty of these rankings. Metformin activates the AMPK pathway to block the NF-κB-mediated inflammatory cascade and modulates chondrocyte autophagy to slow joint degeneration (5, 6, 16). It also provides metabolic benefits—glycemic control and weight reduction—that make it particularly suitable for KOA patients with comorbid type 2 diabetes or metabolic syndrome. Probiotics act through the “gut-joint axis,” modulating gut microbiota homeostasis and reducing the systemic low-grade inflammation triggered by microbial metabolites entering the circulation (3, 7, 11, 13, 15). Because they improve joint inflammation and pain indirectly, without adverse effects on other organ systems, probiotics represent a reasonable alternative for patients with NSAID contraindications. Celecoxib's low safety ranking is consistent with the well-documented gastrointestinal and cardiovascular risks of long-term NSAID use (29), underscoring the importance of adhering to prescribing indications and treatment duration.

### 4.3 Clinical implications

SUCRA values reflect ranking probabilities rather than direct measures of effect size. The clinical suggestions below are based on relative rankings within the network and should not be taken as definitive evidence of superiority. Because the certainty of evidence for most indirect comparisons was low or very low (Table 2), these recommendations should be viewed as preliminary guidance for individualized long-term pharmacological management of KOA:

1. For patients whose primary complaint is moderate-to-severe joint pain and who have no contraindications to NSAID use, celecoxib is a reasonable first choice for rapid pain control and symptom improvement, although the supporting evidence certainty varies (1, 9, 17);
2. When limited joint mobility and functional decline are the primary concerns, or when long-term pain control has been inadequate, krill oil offers a potential option for balancing pain relief with functional improvement, though these findings rest primarily on limited direct evidence (8, 12, 21, 22).
3. For high-risk populations (e.g., elderly patients or those with gastrointestinal or cardiovascular contraindications to NSAIDs), probiotics or metformin are alternatives with favorable safety profiles (5, 7, 10, 11, 16, 28); among these, metformin may be particularly suitable for patients with type 2 diabetes, obesity, or metabolic syndrome, offering dual benefits of metabolic regulation and joint symptom improvement (5, 6, 16).
4. In patients with mild to moderate KOA, CS+GH may be considered for long-term maintenance therapy, though the evidence for chondroprotection remains limited (2, 9, 14, 17, 18, 25).

### 4.4 Study limitations

Several limitations of this study warrant cautious interpretation. Although our search covered database inception to February 2026, some early landmark trials on celecoxib and glucosamine/chondroitin were published before 2000; these were nevertheless captured by our comprehensive search strategy. The limited number of early trials and the evolution of diagnostic criteria and outcome measurement tools over time may contribute to heterogeneity. No direct head-to-head RCTs compared certain interventions (e.g., krill oil vs. metformin or probiotics vs. CS+GH), and several pooled effect sizes were derived from indirect evidence, resulting in lower GRADE ratings for those comparisons (27). This study included only 6-month follow-up data and lacks evidence on efficacy and safety beyond 1 year, making it impossible to assess the long-term benefits and risks of the various interventions. Considerable heterogeneity was present in baseline characteristics (age, disease duration, and Kellgren-Lawrence grade) as well as in intervention dosage and duration, which may have influenced the pooled effect sizes. Methodological information on allocation concealment and blinding was insufficiently reported in some included studies (3, 4, 24), raising concerns about potential selection and measurement bias. The limited number of studies per outcome (range: 4–12 RCTs per network) and inconsistent reporting of subgroup-relevant covariates across trials (e.g., KL grade, symptom duration, and body mass index) precluded meaningful subgroup analyses or meta-regression by age, disease severity, or comorbid conditions. While leave-one-out sensitivity analyses confirmed the robustness of the primary VAS pain findings, the instability observed in some WOMAC subscale comparisons when excluding individual studies highlights the susceptibility of smaller networks to the influence of single trials; additional head-to-head RCTs are needed to validate these exploratory findings. A further limitation of this NMA is the substantial variability in evidence certainty across the 15 comparative pairs. While core traditional regimens yielded acceptable data, 12 of the 15 final network estimates were allocated to low or very low certainty tiers. The extreme statistical heterogeneity observed in certain direct comparisons (e.g., glucosamine or probiotics vs. placebo) further compromised the robustness of the network. Consequently, the lack of high-certainty direct head-to-head evidence limits how broadly these hierarchical rankings can be applied to clinical practice.

### 4.5 Future research directions

Several directions for future research follow from the limitations discussed above. Multicenter, large-sample, double-blind head-to-head RCTs with follow-up exceeding 1 year are needed to generate high-quality direct evidence comparing the long-term efficacy and safety of different interventions. Stratified subgroup analyses should examine whether treatment effectiveness differs by age group, disease duration, severity, and comorbidity profile, enabling more individualized therapy. Combination strategies deserve investigation to determine whether NSAIDs combined with chondroprotectors, probiotics, or metformin produce additive benefits, with the aim of optimizing multi-agent treatment approaches for KOA. Basic mechanistic research should further clarify the roles of the “gut-joint axis” and AMPK pathway in KOA pathogenesis and progression (5–7, 11, 13, 15, 16), potentially identifying new targets and research avenues for targeted KOA treatment.

## 5 Conclusions

This network meta-analysis evaluated the efficacy and safety of five treatment classes—celecoxib, glucosamine and chondroitin, krill oil, probiotics, and metformin—in KOA patients over a treatment period of up to six months. Based on SUCRA rankings, celecoxib was associated with the greatest reductions in joint pain and total symptom burden; krill oil showed potential benefits for joint pain relief and function; and probiotics and metformin demonstrated better tolerability and improved safety profiles. These findings should be interpreted with caution, as most comparative estimates in the network were supported by low- or very-low-certainty evidence (GRADE; Table 2).

Overall, this review identifies clinical scenarios in which certain treatments may be more appropriate and offers preliminary recommendations for clinicians selecting therapies aligned with a patient's primary treatment goals, comorbidities, and drug interactions. The consistency tests, publication bias analyses, and sensitivity checks showed generally stable network behavior, but the underlying evidence for most comparisons was limited: 12 of 15 final network estimates fell into low or very low certainty tiers. Prospective, large-scale, long-term head-to-head RCTs are needed to confirm these exploratory conclusions and refine precision treatment strategies for KOA.

## Data Availability

The original contributions presented in the study are included in the article/Supplementary material, further inquiries can be directed to the corresponding author.
