# Supplementary material for: Network meta-analysis of metformin, probiotics, krill oil, celecoxib, and glucosamine/chondroitin in the treatment of knee osteoarthritis
Source: Front Med (Lausanne). 2026 Jul 22;13:1862817. doi: 10.3389/fmed.2026.1862817 (PMC13437381; doi:10.3389/fmed.2026.1862817)

Treatment	Direct Comparisons	Random Effects Model	MD	95%-CI	P-Score
Placebo	2		0.90	[0.67; 1.13]	0.00
Krill oil	2		-0.17	[-0.41; 0.08]	0.49
Metformin	2		-0.24	[-0.47; -0.01]	0.64
Probiotics	3		-0.26	[-0.48; -0.05]	0.68
Celecoxib	0		-0.48	[-0.78; -0.17]	0.97

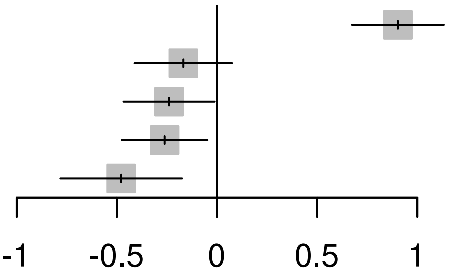

Supplement: Supplementary file 1 [file Data_Sheet_1.zip › S1 file Summary table of MD values and 95% CIs for VAS pain scores across interventions/VAS Pain (6 Months)-forestplot.pdf]
