# Supplementary material for: Network meta-analysis of metformin, probiotics, krill oil, celecoxib, and glucosamine/chondroitin in the treatment of knee osteoarthritis
Source: Front Med (Lausanne). 2026 Jul 22;13:1862817. doi: 10.3389/fmed.2026.1862817 (PMC13437381; doi:10.3389/fmed.2026.1862817)
Supplement: Supplementary file 1 [file Data_Sheet_1.zip › S1 file Summary table of MD values and 95% CIs for VAS pain scores across interventions/VAS Pain (6 Months)-netgraph.pdf]

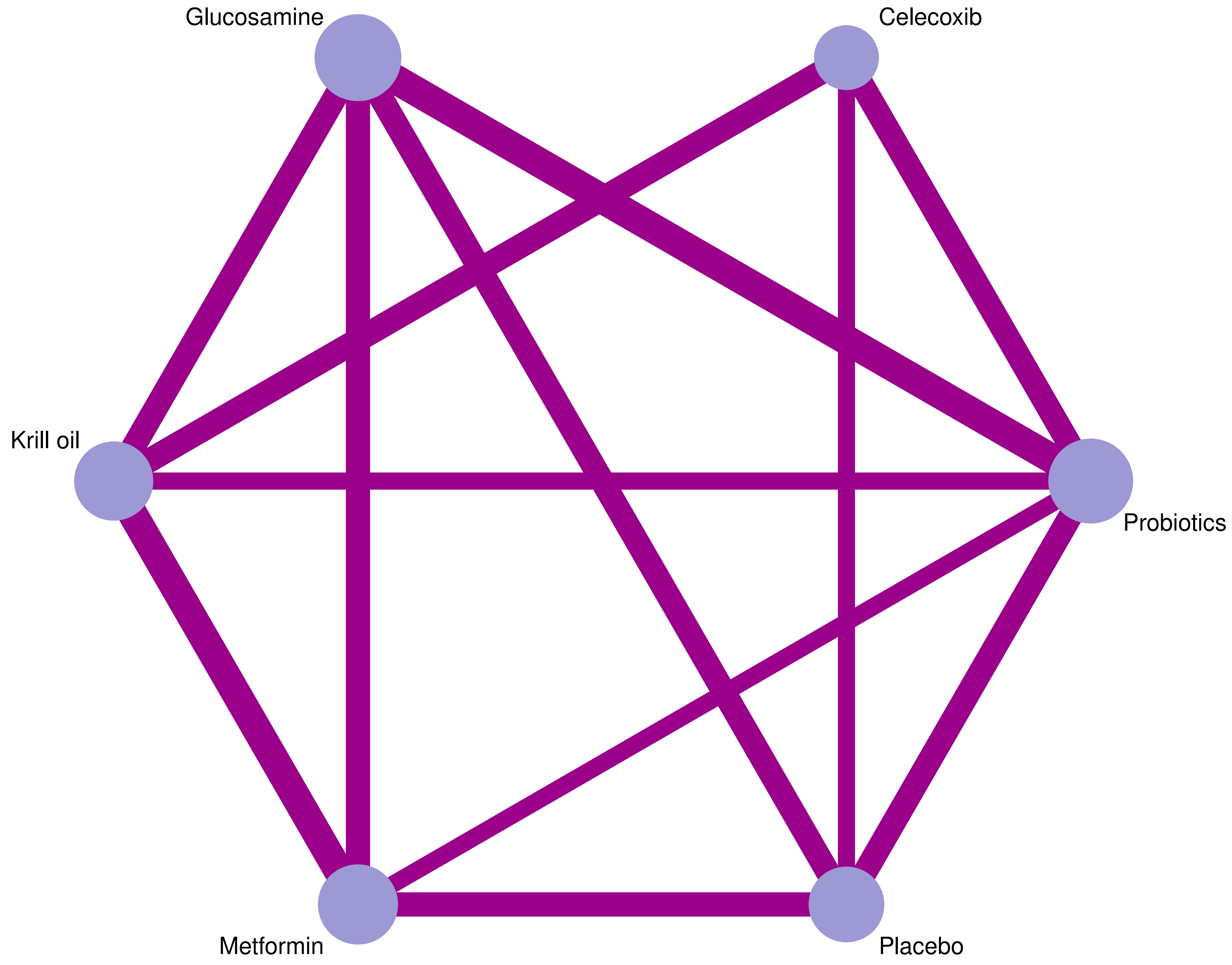
Glucosamine
Celecoxib
Krill oil
Probiotics
Metformin
Placebo
