# Supplementary material for: Network meta-analysis of metformin, probiotics, krill oil, celecoxib, and glucosamine/chondroitin in the treatment of knee osteoarthritis
Source: Front Med (Lausanne). 2026 Jul 22;13:1862817. doi: 10.3389/fmed.2026.1862817 (PMC13437381; doi:10.3389/fmed.2026.1862817)
Supplement: Supplementary file 1 [file Data_Sheet_1.zip › S1 file Summary table of MD values and 95% CIs for VAS pain scores across interventions/VAS Pain (6 Months)-pairwise forestplot.pdf]

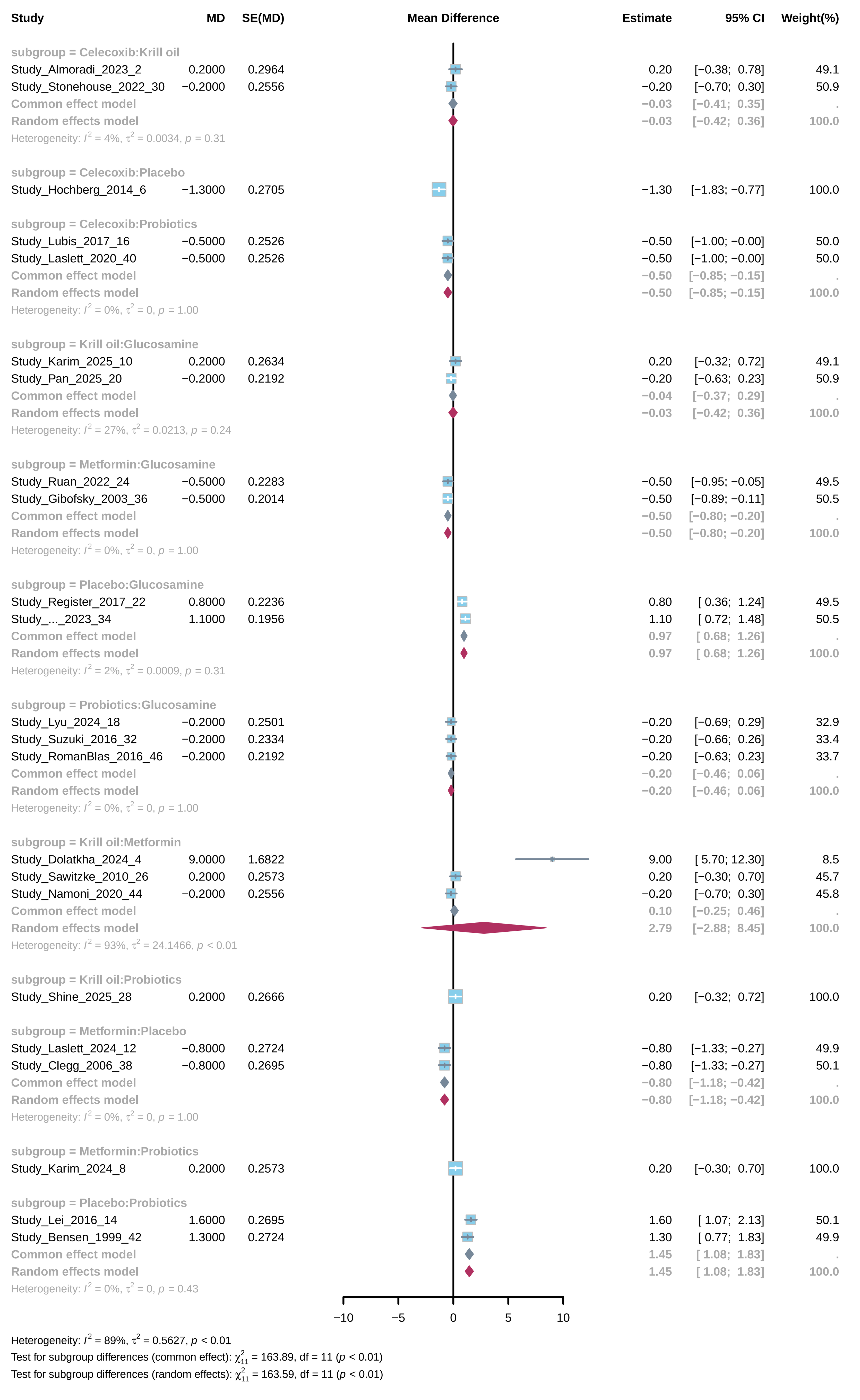
Study MD SE(MD) Mean Difference Estimate 95% CI Weight(%)
subgroup = Celecoxib:Krill oil
Study_Almoradi_2023_2 0.2000 0.2964 0.20 [−0.38; 0.78] 49.1
Study_Stonehouse_2022_30 −0.2000 0.2556 −0.20 [−0.70; 0.30] 50.9
Common effect model −0.03 [−0.41; 0.35] .
Random effects model −0.03 [−0.42; 0.36] 100.0
Heterogeneity: I^2 = 4%, τ^2 = 0.0034, p = 0.31
subgroup = Celecoxib:Placebo
Study_Hochberg_2014_6 −1.3000 0.2705 −1.30 [−1.83; −0.77] 100.0
subgroup = Celecoxib:Probiotics
Study_Lubis_2017_16 −0.5000 0.2526 −0.50 [−1.00; −0.00] 50.0
Study_Laslett_2020_40 −0.5000 0.2526 −0.50 [−1.00; −0.00] 50.0
Common effect model −0.50 [−0.85; −0.15] .
Random effects model −0.50 [−0.85; −0.15] 100.0
Heterogeneity: I^2 = 0%, τ^2 = 0, p = 1.00
subgroup = Krill oil:Glucosamine
Study_Karim_2025_10 0.2000 0.2634 0.20 [−0.32; 0.72] 49.1
Study_Pan_2025_20 −0.2000 0.2192 −0.20 [−0.63; 0.23] 50.9
Common effect model −0.04 [−0.37; 0.29] .
Random effects model −0.03 [−0.42; 0.36] 100.0
Heterogeneity: I^2 = 27%, τ^2 = 0.0213, p = 0.24
subgroup = Metformin:Glucosamine
Study_Ruan_2022_24 −0.5000 0.2283 −0.50 [−0.95; −0.05] 49.5
Study_Gibofsky_2003_36 −0.5000 0.2014 −0.50 [−0.89; −0.11] 50.5
Common effect model −0.50 [−0.80; −0.20] .
Random effects model −0.50 [−0.80; −0.20] 100.0
Heterogeneity: I^2 = 0%, τ^2 = 0, p = 1.00
subgroup = Placebo:Glucosamine
Study_Register_2017_22 0.8000 0.2236 0.80 [0.36; 1.24] 49.5
Study_..._2023_34 1.1000 0.1956 1.10 [0.72; 1.48] 50.5
Common effect model 0.97 [0.68; 1.26] .
Random effects model 0.97 [0.68; 1.26] 100.0
Heterogeneity: I^2 = 2%, τ^2 = 0.0009, p = 0.31
subgroup = Probiotics:Glucosamine
Study_Lyu_2024_18 −0.2000 0.2501 −0.20 [−0.69; 0.29] 32.9
Study_Suzuki_2016_32 −0.2000 0.2334 −0.20 [−0.66; 0.26] 33.4
Study_RomanBlas_2016_46 −0.2000 0.2192 −0.20 [−0.63; 0.23] 33.7
Common effect model −0.20 [−0.46; 0.06] .
Random effects model −0.20 [−0.46; 0.06] 100.0
Heterogeneity: I^2 = 0%, τ^2 = 0, p = 1.00
subgroup = Krill oil:Metformin
Study_Dolatkha_2024_4 9.0000 1.6822 9.00 [5.70; 12.30] 8.5
Study_Sawitzke_2010_26 0.2000 0.2573 0.20 [−0.30; 0.70] 45.7
Study_Namoni_2020_44 −0.2000 0.2556 −0.20 [−0.70; 0.30] 45.8
Common effect model 0.10 [−0.25; 0.46] .
Random effects model 2.79 [−2.88; 8.45] 100.0
Heterogeneity: I^2 = 93%, τ^2 = 24.1466, p < 0.01
subgroup = Krill oil:Probiotics
Study_Shine_2025_28 0.2000 0.2666 0.20 [−0.32; 0.72] 100.0
subgroup = Metformin:Placebo
Study_Laslett_2024_12 −0.8000 0.2724 −0.80 [−1.33; −0.27] 49.9
Study_Clegg_2006_38 −0.8000 0.2695 −0.80 [−1.33; −0.27] 50.1
Common effect model −0.80 [−1.18; −0.42] .
Random effects model −0.80 [−1.18; −0.42] 100.0
Heterogeneity: I^2 = 0%, τ^2 = 0, p = 1.00
subgroup = Metformin:Probiotics
Study_Karim_2024_8 0.2000 0.2573 0.20 [−0.30; 0.70] 100.0
subgroup = Placebo:Probiotics
Study_Lei_2016_14 1.6000 0.2695 1.60 [1.07; 2.13] 50.1
Study_Bensen_1999_42 1.3000 0.2724 1.30 [0.77; 1.83] 49.9
Common effect model 1.45 [1.08; 1.83] .
Random effects model 1.45 [1.08; 1.83] 100.0
Heterogeneity: I^2 = 0%, τ^2 = 0, p = 0.43
−10 −5 0 5 10
Heterogeneity: I^2 = 89%, τ^2 = 0.5627, p < 0.01
Test for subgroup differences (common effect): χ^2_{11} = 163.89, df = 11 (p < 0.01)
Test for subgroup differences (random effects): χ^2_{11} = 163.59, df = 11 (p < 0.01)
