# Supplementary material for: Network meta-analysis of metformin, probiotics, krill oil, celecoxib, and glucosamine/chondroitin in the treatment of knee osteoarthritis
Source: Front Med (Lausanne). 2026 Jul 22;13:1862817. doi: 10.3389/fmed.2026.1862817 (PMC13437381; doi:10.3389/fmed.2026.1862817)

Comparison	Number of Studies	Direct Evidence	I2	MD	95%-CI
**Celecoxib:Glucosamine**					
Direct estimate	0	0			
Indirect estimate				-0.48	[-0.78; -0.17]
Network estimate				-0.48	[-0.78; -0.17]
**Celecoxib:Krill oil**					
Direct estimate	2	0.52	4%	-0.03	[-0.43; 0.38]
Indirect estimate				-0.62	[-1.04; -0.19]
Network estimate				-0.31	[-0.60; -0.02]
**Celecoxib:Metformin**					
Direct estimate	0	0			
Indirect estimate				-0.24	[-0.57; 0.09]
Network estimate				-0.24	[-0.57; 0.09]
**Celecoxib:Placebo**					
Direct estimate	1	0.30		-1.30	[-1.87; -0.73]
Indirect estimate				-1.42	[-1.79; -1.04]
Network estimate				-1.38	[-1.69; -1.07]
**Celecoxib:Probiotics**					
Direct estimate	2	0.54	0%	-0.50	[-0.88; -0.12]
Indirect estimate				0.11	[-0.30; 0.52]
Network estimate				-0.22	[-0.50; 0.06]
**Krill oil:Glucosamine**					
Direct estimate	2	0.45	27%	-0.03	[-0.39; 0.33]
Indirect estimate				-0.28	[-0.61; 0.05]
Network estimate				-0.17	[-0.41; 0.08]
**Metformin:Glucosamine**					
Direct estimate	2	0.47	0%	-0.50	[-0.83; -0.17]
Indirect estimate				-0.01	[-0.32; 0.31]
Network estimate				-0.24	[-0.47; -0.01]
**Placebo:Glucosamine**					
Direct estimate	2	0.50	2%	0.97	[0.64; 1.29]
Indirect estimate				0.84	[0.52; 1.16]
Network estimate				0.90	[0.67; 1.13]
**Probiotics:Glucosamine**					
Direct estimate	3	0.54	0%	-0.20	[-0.49; 0.09]
Indirect estimate				-0.33	[-0.65; -0.02]
Network estimate				-0.26	[-0.48; -0.05]
**Krill oil:Metformin**					
Direct estimate	3	0.46	93%	0.12	[-0.26; 0.50]
Indirect estimate				0.03	[-0.32; 0.38]
Network estimate				0.07	[-0.19; 0.33]
**Krill oil:Placebo**					
Direct estimate	0	0			
Indirect estimate				-1.07	[-1.36; -0.78]
Network estimate				-1.07	[-1.36; -0.78]
**Krill oil:Probiotics**					
Direct estimate	1	0.22		0.20	[-0.36; 0.76]
Indirect estimate				0.06	[-0.23; 0.36]
Network estimate				0.09	[-0.17; 0.36]
**Metformin:Placebo**					
Direct estimate	2	0.41	0%	-0.80	[-1.20; -0.40]
Indirect estimate				-1.38	[-1.71; -1.04]
Network estimate				-1.14	[-1.40; -0.89]
**Metformin:Probiotics**					
Direct estimate	1	0.22		0.20	[-0.35; 0.75]
Indirect estimate				-0.03	[-0.32; 0.26]
Network estimate				0.02	[-0.24; 0.28]
**Placebo:Probiotics**					
Direct estimate	2	0.37	0%	1.45	[1.05; 1.85]
Indirect estimate				1.00	[0.69; 1.31]
Network estimate				1.16	[0.92; 1.41]

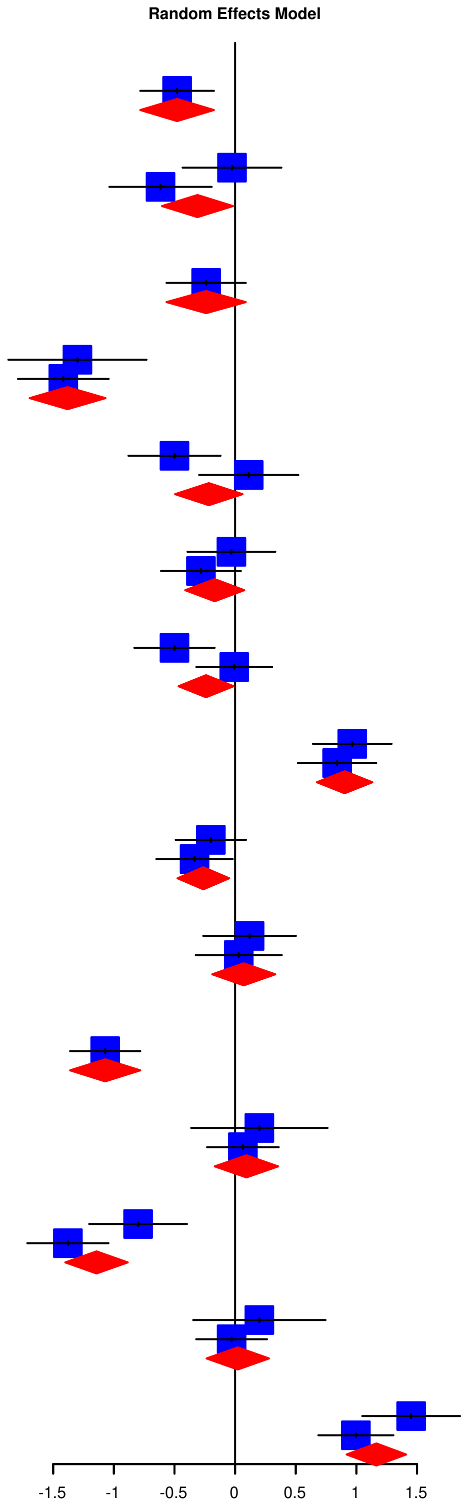

Supplement: Supplementary file 1 [file Data_Sheet_1.zip › S1 file Summary table of MD values and 95% CIs for VAS pain scores across interventions/VAS Pain (6 Months)-random-nodesplit.pdf]
