# Supplementary material for: Network meta-analysis of metformin, probiotics, krill oil, celecoxib, and glucosamine/chondroitin in the treatment of knee osteoarthritis
Source: Front Med (Lausanne). 2026 Jul 22;13:1862817. doi: 10.3389/fmed.2026.1862817 (PMC13437381; doi:10.3389/fmed.2026.1862817)
Supplement: Supplementary file 1 [file Data_Sheet_1.zip › S2 file Summary table of MD values and 95% CIs for WOMAC total scores across interventions/WOMAC Total Score (6 Months)-pairwise forestplot.pdf]

Study	MD	SE(MD)	Estimate	95% CI	Weight(%)
subgroup = Celecoxib:Probiotics					
Probiotics improve functional perfor SO Eur J Nutr_6	−0.3000	0.1914	−0.30	[−0.68; 0.08]	100.0
subgroup = Glucosamine:Placebo					
.. + ... 1500+1200.PDF_10	−5.3000	0.3874	−5.30	[−6.06; −4.54]	50.5
.. + ... 1500+1200.PDF_16	−0.9500	0.4537	−0.95	[−1.84; −0.06]	49.5
**Common effect model**			**−3.47**	**[−4.04; −2.89]**	**.**
**Random effects model**			**−3.13**	**[−7.39; 1.13]**	**100.0**
Heterogeneity: $I^2 = 98\%$, $\tau^2 = 9.2833$, $p < 0.01$					
subgroup = Krill oil:Placebo					
Krill oil improved osteoarthritic kn Source Am J C_4	−1.3000	0.2695	−1.30	[−1.83; −0.77]	50.0
Krill oil improved osteoarthritic kn Source Am J C_18	−0.8000	0.2724	−0.80	[−1.33; −0.27]	50.0
**Common effect model**			**−1.05**	**[−1.43; −0.68]**	**.**
**Random effects model**			**−1.05**	**[−1.54; −0.56]**	**100.0**
Heterogeneity: $I^2 = 41\%$, $\tau^2 = 0.0516$, $p = 0.19$					
subgroup = Metformin:Probiotics					
Probiotics improve functional perfor SO Eur J Nutr_8	−1.0000	0.1914	−1.00	[−1.38; −0.62]	100.0
subgroup = Probiotics:Placebo					
Probiotics improve functional perfor SO Eur J Nutr_12	0.1000	0.2101	0.10	[−0.31; 0.51]	50.0
Probiotics improve functional perfor SO Eur J Nutr_20	−1.4500	0.2124	−1.45	[−1.87; −1.03]	50.0
**Common effect model**			**−0.67**	**[−0.96; −0.37]**	**.**
**Random effects model**			**−0.67**	**[−2.19; 0.84]**	**100.0**
Heterogeneity: $I^2 = 96\%$, $\tau^2 = 1.1566$, $p < 0.01$					

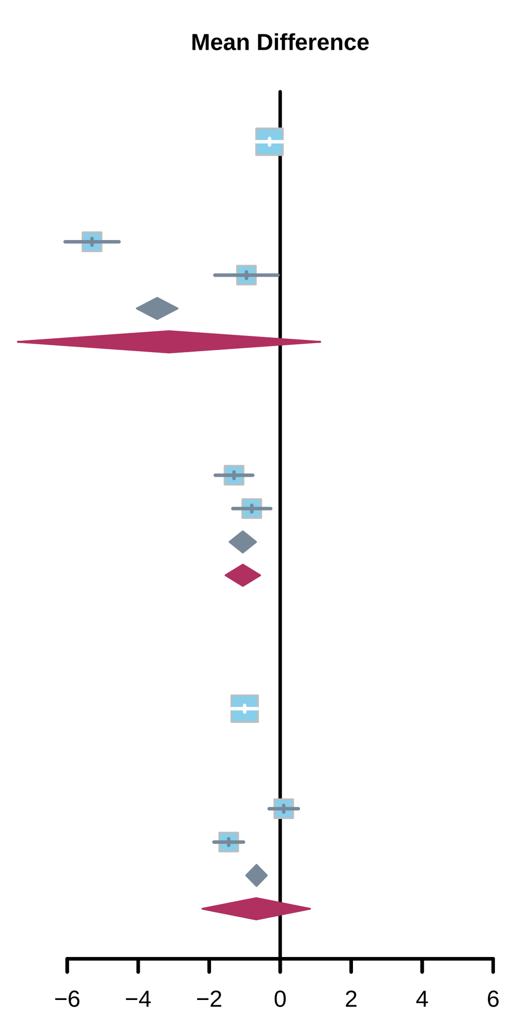

Heterogeneity: $I^2 = 96\%$, $\tau^2 = 2.5943$, $p < 0.01$
Test for subgroup differences (common effect): $\chi^2_4 = 88.34$, df = 4 ($p < 0.01$)
Test for subgroup differences (random effects): $\chi^2_4 = 9.90$, df = 4 ($p = 0.04$)
