# Supplementary material for: Network meta-analysis of metformin, probiotics, krill oil, celecoxib, and glucosamine/chondroitin in the treatment of knee osteoarthritis
Source: Front Med (Lausanne). 2026 Jul 22;13:1862817. doi: 10.3389/fmed.2026.1862817 (PMC13437381; doi:10.3389/fmed.2026.1862817)

Comparison	Number of Studies	Direct Evidence	I2	MD	95%-CI
**Celecoxib:Glucosamine**					
Direct estimate	0	0			
Indirect estimate				2.17	[-2.98; 7.31]
Network estimate				2.17	[-2.98; 7.31]
**Celecoxib:Krill oil**					
Direct estimate	0	0			
Indirect estimate				0.08	[-5.05; 5.20]
Network estimate				0.08	[-5.05; 5.20]
**Celecoxib:Metformin**					
Direct estimate	0	0			
Indirect estimate				0.70	[-4.42; 5.82]
Network estimate				0.70	[-4.42; 5.82]
**Celecoxib:Placebo**					
Direct estimate	0	0			
Indirect estimate				-0.97	[-5.41; 3.46]
Network estimate				-0.97	[-5.41; 3.46]
**Celecoxib:Probiotics**					
Direct estimate	1	1.00		-0.30	[-3.92; 3.32]
Indirect estimate					
Network estimate				-0.30	[-3.92; 3.32]
**Glucosamine:Krill oil**					
Direct estimate	0	0			
Indirect estimate				-2.09	[-5.76; 1.57]
Network estimate				-2.09	[-5.76; 1.57]
**Glucosamine:Metformin**					
Direct estimate	0	0			
Indirect estimate				-1.47	[-6.61; 3.68]
Network estimate				-1.47	[-6.61; 3.68]
**Glucosamine:Placebo**					
Direct estimate	2	1.00	98%	-3.14	[-5.75; -0.53]
Indirect estimate					
Network estimate				-3.14	[-5.75; -0.53]
**Glucosamine:Probiotics**					
Direct estimate	0	0			
Indirect estimate				-2.47	[-6.12; 1.19]
Network estimate				-2.47	[-6.12; 1.19]
**Krill oil:Metformin**					
Direct estimate	0	0			
Indirect estimate				0.62	[-4.50; 5.75]
Network estimate				0.62	[-4.50; 5.75]
**Krill oil:Placebo**					
Direct estimate	2	1.00	41%	-1.05	[-3.62; 1.52]
Indirect estimate					
Network estimate				-1.05	[-3.62; 1.52]
**Krill oil:Probiotics**					
Direct estimate	0	0			
Indirect estimate				-0.38	[-4.00; 3.25]
Network estimate				-0.38	[-4.00; 3.25]
**Metformin:Placebo**					
Direct estimate	0	0			
Indirect estimate				-1.67	[-6.11; 2.76]
Network estimate				-1.67	[-6.11; 2.76]
**Metformin:Probiotics**					
Direct estimate	1	1.00		-1.00	[-4.62; 2.62]
Indirect estimate					
Network estimate				-1.00	[-4.62; 2.62]
**Probiotics:Placebo**					
Direct estimate	2	1.00	96%	-0.67	[-3.24; 1.89]
Indirect estimate					
Network estimate				-0.67	[-3.24; 1.89]

Random Effects Model

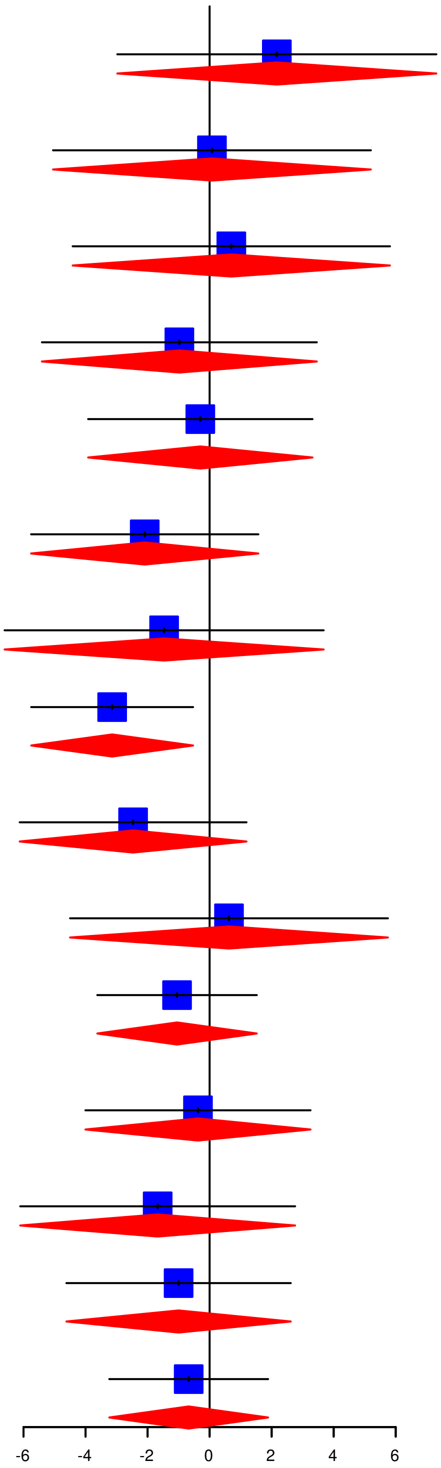

Supplement: Supplementary file 1 [file Data_Sheet_1.zip › S2 file Summary table of MD values and 95% CIs for WOMAC total scores across interventions/WOMAC Total Score (6 Months)-random-nodesplit.pdf]
