# Supplementary material for: Network meta-analysis of metformin, probiotics, krill oil, celecoxib, and glucosamine/chondroitin in the treatment of knee osteoarthritis
Source: Front Med (Lausanne). 2026 Jul 22;13:1862817. doi: 10.3389/fmed.2026.1862817 (PMC13437381; doi:10.3389/fmed.2026.1862817)
Supplement: Supplementary file 1 [file Data_Sheet_1.zip › S3 file Summary table of MD values and 95% CIs for WOMAC pain subscale scores across/WOMAC Pain Score at 6 Months-random-nodesplit.pdf]

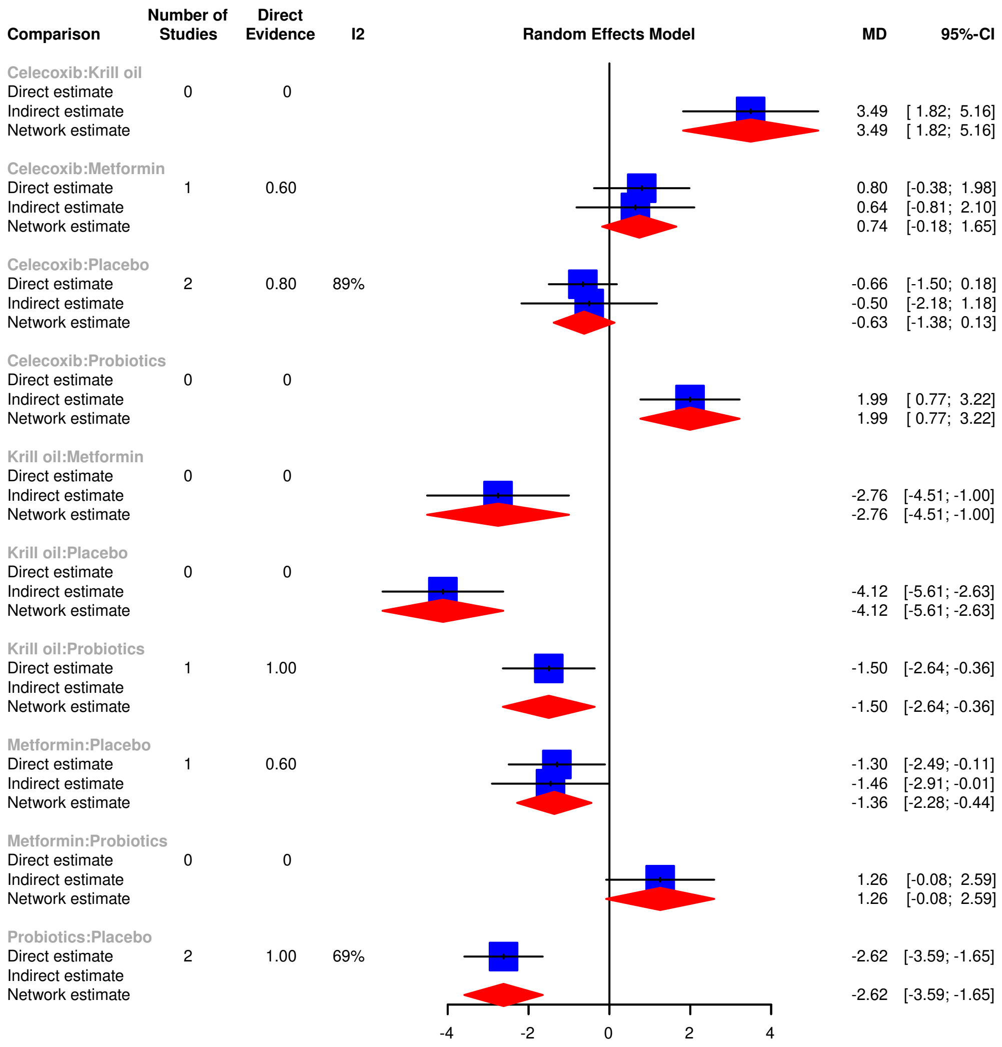
Comparison
Number of Studies
Direct Evidence
I2
Random Effects Model
MD
95%-CI
Celecoxib:Krill oil
Direct estimate 0 0
Indirect estimate 3.49 [1.82; 5.16]
Network estimate 3.49 [1.82; 5.16]
Celecoxib:Metformin
Direct estimate 1 0.60 0.80 [-0.38; 1.98]
Indirect estimate 0.64 [-0.81; 2.10]
Network estimate 0.74 [-0.18; 1.65]
Celecoxib:Placebo
Direct estimate 2 0.80 89% -0.66 [-1.50; 0.18]
Indirect estimate -0.50 [-2.18; 1.18]
Network estimate -0.63 [-1.38; 0.13]
Celecoxib:Probiotics
Direct estimate 0 0
Indirect estimate 1.99 [0.77; 3.22]
Network estimate 1.99 [0.77; 3.22]
Krill oil:Metformin
Direct estimate 0 0
Indirect estimate -2.76 [-4.51; -1.00]
Network estimate -2.76 [-4.51; -1.00]
Krill oil:Placebo
Direct estimate 0 0
Indirect estimate -4.12 [-5.61; -2.63]
Network estimate -4.12 [-5.61; -2.63]
Krill oil:Probiotics
Direct estimate 1 1.00 -1.50 [-2.64; -0.36]
Indirect estimate
Network estimate -1.50 [-2.64; -0.36]
Metformin:Placebo
Direct estimate 1 0.60 -1.30 [-2.49; -0.11]
Indirect estimate -1.46 [-2.91; -0.01]
Network estimate -1.36 [-2.28; -0.44]
Metformin:Probiotics
Direct estimate 0 0
Indirect estimate 1.26 [-0.08; 2.59]
Network estimate 1.26 [-0.08; 2.59]
Probiotics:Placebo
Direct estimate 2 1.00 69% -2.62 [-3.59; -1.65]
Indirect estimate
Network estimate -2.62 [-3.59; -1.65]
-4
-2
0
2
4
