# Supplementary material for: Network meta-analysis of metformin, probiotics, krill oil, celecoxib, and glucosamine/chondroitin in the treatment of knee osteoarthritis
Source: Front Med (Lausanne). 2026 Jul 22;13:1862817. doi: 10.3389/fmed.2026.1862817 (PMC13437381; doi:10.3389/fmed.2026.1862817)
Supplement: Supplementary file 1 [file Data_Sheet_1.zip › S4 file Summary table of MD values and 95% CIs for WOMAC functional subscale scores across/WOMAC Function at 6 Months-funnel plot.pdf]

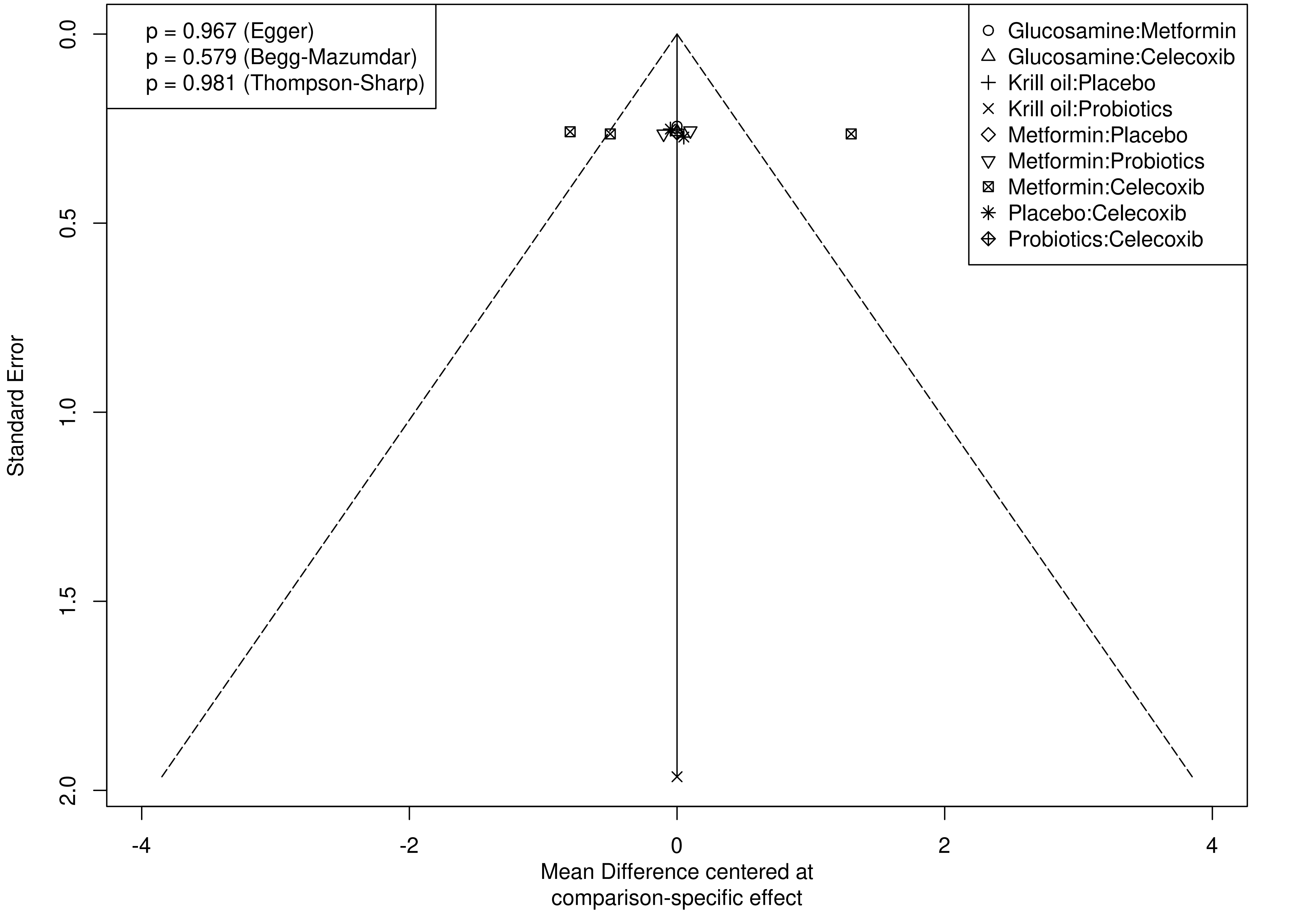
p = 0.967 (Egger)
p = 0.579 (Begg-Mazumdar)
p = 0.981 (Thompson-Sharp)
Glucosamine:Metformin
Glucosamine:Celecoxib
Krill oil:Placebo
Krill oil:Probiotics
Metformin:Placebo
Metformin:Probiotics
Metformin:Celecoxib
Placebo:Celecoxib
Probiotics:Celecoxib
Standard Error
0.0
0.5
1.0
1.5
2.0
-4
-2
0
2
4
Mean Difference centered at
comparison-specific effect
