# Supplementary material for: Network meta-analysis of metformin, probiotics, krill oil, celecoxib, and glucosamine/chondroitin in the treatment of knee osteoarthritis
Source: Front Med (Lausanne). 2026 Jul 22;13:1862817. doi: 10.3389/fmed.2026.1862817 (PMC13437381; doi:10.3389/fmed.2026.1862817)
Supplement: Supplementary file 1 [file Data_Sheet_1.zip › S4 file Summary table of MD values and 95% CIs for WOMAC functional subscale scores across/WOMAC Function at 6 Months-random-nodesplit.pdf]

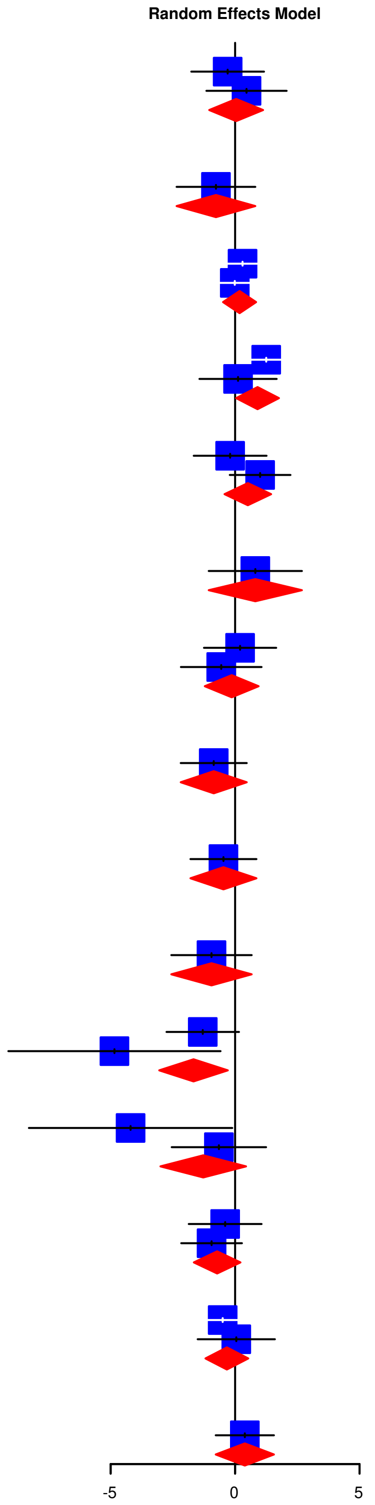

Comparison	Number of Studies	Direct Evidence	I2	MD	95%-CI
**Glucosamine:Celecoxib**					
Direct estimate	1	0.55		-0.30	[-1.76; 1.16]
Indirect estimate				0.46	[-1.15; 2.07]
Network estimate				0.04	[-1.04; 1.12]
**Krill oil:Celecoxib**					
Direct estimate	0	0			
Indirect estimate				-0.77	[-2.35; 0.81]
Network estimate				-0.77	[-2.35; 0.81]
**Metformin:Celecoxib**					
Direct estimate	3	0.62	95%	0.30	[-0.55; 1.14]
Indirect estimate				-0.01	[-1.09; 1.06]
Network estimate				0.18	[-0.49; 0.84]
**Placebo:Celecoxib**					
Direct estimate	2	0.69	0%	1.25	[0.21; 2.28]
Indirect estimate				0.12	[-1.43; 1.67]
Network estimate				0.90	[0.04; 1.76]
**Probiotics:Celecoxib**					
Direct estimate	1	0.41		-0.20	[-1.66; 1.26]
Indirect estimate				1.00	[-0.21; 2.22]
Network estimate				0.51	[-0.43; 1.45]
**Glucosamine:Krill oil**					
Direct estimate	0	0			
Indirect estimate				0.81	[-1.06; 2.68]
Network estimate				0.81	[-1.06; 2.68]
**Glucosamine:Metformin**					
Direct estimate	1	0.56		0.20	[-1.25; 1.65]
Indirect estimate				-0.56	[-2.18; 1.06]
Network estimate				-0.14	[-1.22; 0.94]
**Glucosamine:Placebo**					
Direct estimate	0	0			
Indirect estimate				-0.86	[-2.19; 0.47]
Network estimate				-0.86	[-2.19; 0.47]
**Glucosamine:Probiotics**					
Direct estimate	0	0			
Indirect estimate				-0.47	[-1.79; 0.86]
Network estimate				-0.47	[-1.79; 0.86]
**Krill oil:Metformin**					
Direct estimate	0	0			
Indirect estimate				-0.95	[-2.56; 0.67]
Network estimate				-0.95	[-2.56; 0.67]
**Krill oil:Placebo**					
Direct estimate	1	0.90		-1.30	[-2.76; 0.16]
Indirect estimate				-4.85	[-9.11; -0.59]
Network estimate				-1.67	[-3.05; -0.29]
**Krill oil:Probiotics**					
Direct estimate	1	0.18		-4.20	[-8.29; -0.11]
Indirect estimate				-0.65	[-2.55; 1.25]
Network estimate				-1.28	[-3.00; 0.44]
**Metformin:Placebo**					
Direct estimate	1	0.41		-0.40	[-1.86; 1.06]
Indirect estimate				-0.95	[-2.17; 0.27]
Network estimate				-0.72	[-1.66; 0.22]
**Metformin:Probiotics**					
Direct estimate	2	0.69	0%	-0.50	[-1.53; 0.53]
Indirect estimate				0.05	[-1.50; 1.60]
Network estimate				-0.33	[-1.19; 0.53]
**Placebo:Probiotics**					
Direct estimate	0	0			
Indirect estimate				0.39	[-0.78; 1.56]
Network estimate				0.39	[-0.78; 1.56]
