# Supplementary material for: Network meta-analysis of metformin, probiotics, krill oil, celecoxib, and glucosamine/chondroitin in the treatment of knee osteoarthritis
Source: Front Med (Lausanne). 2026 Jul 22;13:1862817. doi: 10.3389/fmed.2026.1862817 (PMC13437381; doi:10.3389/fmed.2026.1862817)

Comparison	Number of Studies	Direct Evidence	I2	RR	95%-CI
**Celecoxib:CS+GH**					
Direct estimate	2	1.00	73%	0.78	[0.45; 1.36]
Indirect estimate					
Network estimate				0.78	[0.45; 1.36]
**Celecoxib:krill oil**					
Direct estimate	0	0			
Indirect estimate				1.44	[0.61; 3.44]
Network estimate				1.44	[0.61; 3.44]
**Celecoxib:metformin**					
Direct estimate	0	0			
Indirect estimate				0.90	[0.34; 2.39]
Network estimate				0.90	[0.34; 2.39]
**Celecoxib:placebo**					
Direct estimate	2	1.00	57%	1.38	[0.82; 2.34]
Indirect estimate					
Network estimate				1.38	[0.82; 2.34]
**Celecoxib:probiotics**					
Direct estimate	0	0			
Indirect estimate				2.14	[0.32; 14.51]
Network estimate				2.14	[0.32; 14.51]
**CS+GH:krill oil**					
Direct estimate	0	0			
Indirect estimate				1.84	[0.66; 5.15]
Network estimate				1.84	[0.66; 5.15]
**CS+GH:metformin**					
Direct estimate	0	0			
Indirect estimate				1.15	[0.37; 3.53]
Network estimate				1.15	[0.37; 3.53]
**CS+GH:placebo**					
Direct estimate	0	0			
Indirect estimate				1.76	[0.82; 3.79]
Network estimate				1.76	[0.82; 3.79]
**CS+GH:probiotics**					
Direct estimate	0	0			
Indirect estimate				2.73	[0.37; 20.00]
Network estimate				2.73	[0.37; 20.00]
**krill oil:metformin**					
Direct estimate	0	0			
Indirect estimate				0.62	[0.21; 1.83]
Network estimate				0.62	[0.21; 1.83]
**krill oil:placebo**					
Direct estimate	1	1.00		0.96	[0.48; 1.91]
Indirect estimate					
Network estimate				0.96	[0.48; 1.91]
**krill oil:probiotics**					
Direct estimate	0	0			
Indirect estimate				1.48	[0.21; 10.58]
Network estimate				1.48	[0.21; 10.58]
**metformin:placebo**					
Direct estimate	1	1.00		1.53	[0.67; 3.49]
Indirect estimate					
Network estimate				1.53	[0.67; 3.49]
**metformin:probiotics**					
Direct estimate	0	0			
Indirect estimate				2.37	[0.32; 17.79]
Network estimate				2.37	[0.32; 17.79]
**probiotics:placebo**					
Direct estimate	1	1.00		0.65	[0.10; 4.06]
Indirect estimate					
Network estimate				0.65	[0.10; 4.06]

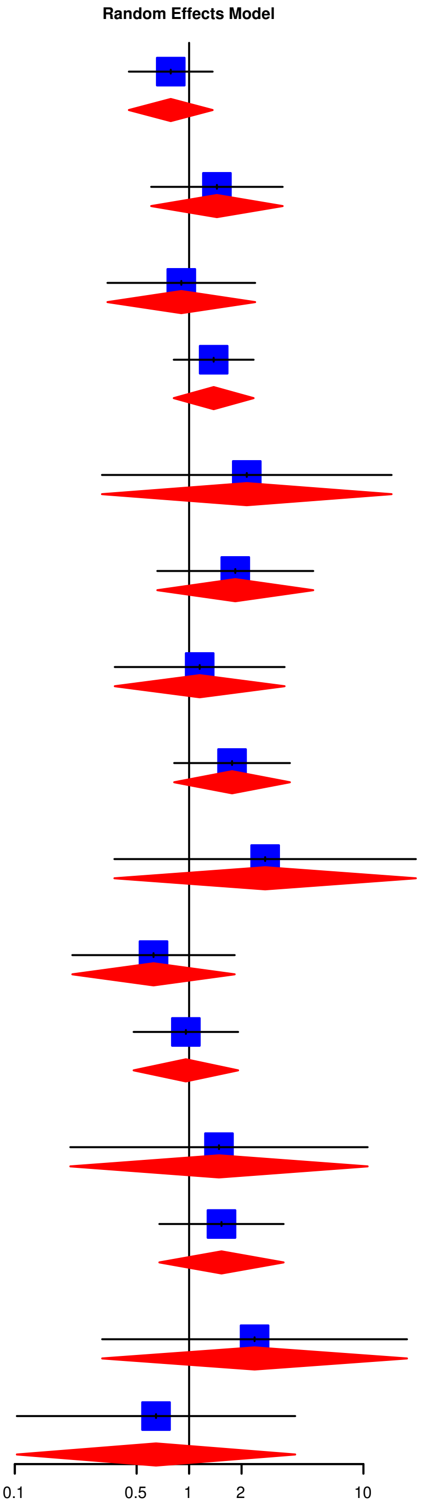

Supplement: Supplementary file 1 [file Data_Sheet_1.zip › S5 file Summary table of RR values and 95% CIs for adverse event incidence across/Safety outcomes-random-nodesplit.pdf]
